# Supplementary material for: A dicarbonate solvent electrolyte for high performance 5 V-Class Lithium-based batteries
Source: Nat Commun. 2024 Jan 15;15:536. doi: 10.1038/s41467-024-44858-3 (PMC10789778; doi:10.1038/s41467-024-44858-3)
Supplement: Supplementary file 1 — Supplementary Information [file 41467_2024_44858_MOESM1_ESM.pdf]

Supplementary information for

A Dicarbonate Solvent Electrolyte for High Performance 5 V-Class Lithium-based Batteries

Xiaozhe Zhang[‡,1], Pan Xu[‡,2], Jianing Duan[‡,2], Xiaodong Lin[‡,1,*], Juanjuan Sun[2], Wenjie Shi[3], Hewei Xu[1], Wenjie Dou[2], Qingyi Zheng[2], Ruming Yuan[2], Jiande Wang[1], Yan Zhang[1], Shanshan Yu[2], Zehan Chen[1], Mingsen Zheng[2], Jean-François Gohy[1], Quanfeng Dong[2,*], Alexandru Vlad[1,*]

[1]Institute of Condensed Matter and Nanosciences, Molecular Chemistry, Materials and Catalysis, Université catholique de Louvain, Louvain-la-Neuve B-1348, Belgium.

[2]Collaborative Innovation Center of Chemistry for Energy Materials (*i*ChEM), State Key Laboratory of Physical Chemistry of Solid Surfaces, Department of Chemistry, College of Chemistry and Chemical Engineering, Engineering Research Centre of Electrochemical Technologies of Ministry of Education, Innovation Laboratory for Sciences and Technologies of Energy Materials of Fujian Province (IKKEM), Xiamen University, Xiamen 361005, China.

[3]Institute for New Energy Materials & Low Carbon Technologies, School of Material Science & Engineering, School of Chemistry & Chemical Engineering, Tianjin University of Technology, Tianjin 300384, China.

‡These authors contributed equally to this work.

*Correspondence: xiaodong.lin@uclouvain.be; qfdong@xmu.edu.cn; alexandru.vlad@uclouvain.be

Supplementary Table 1. Performance comparison of Li||Cu cells in conventional, additive-free single-solvent carbonate-based electrolytes.

Electrolytes	Current density	Capacity	Cycling performance	Ref.
**1 M $LiPF_6$ in DMDOHD**	0.5 mA cm^{-2}	1 mAh cm^{-2}	average CE ~92%<br>> 250 cycles	This work
	1 mA cm^{-2}	1 mAh cm^{-2}	average CE ~87%<br>> 150 cycles	
**1 M $LiPF_6$ in EC**	0.5 mA cm^{-2}	1 mAh cm^{-2}	CE ~40%<br>Dead at ~100 cycles	1
**1 M $LiPF_6$ in DMC**	0.5 mA cm^{-2}	1 mAh cm^{-2}	CE ~20%<br>~100 cycles	
**1 M $LiPF_6$ in DEC**	0.5 mA cm^{-2}	1 mAh cm^{-2}	CE ~5%<br>Dead at ~20 cycles	
**1 M $LiPF_6$ in EMC**	0.5 mA cm^{-2}	1 mAh cm^{-2}	CE ~1.3%<br>Dead at ~50 cycles	
**1 M $LiPF_6$ in PC**	0.2 mA cm^{-2}	0.4 mAh cm^{-2}	CE ~73.2%<br>10 cycles	2

Supplementary Table 2. Performance comparison of Li||$LiNi_{0.5}Mn_{1.5}O_4$ cells in conventional additive-free single-solvent carbonate-based electrolytes.

Electrolytes	Voltage (V vs. Li/Li^+)	Cycling condition	Capacity retention	Ref.
**1 M $LiPF_6$ in DMDOHD**	3.0–4.9 V	C/5 at R.T.	~94% after 200 cycles	This work
		C/3 at R.T.	~94% after 250 cycles	
**1.3 M $LiPF_6$ in EC/EMC/DEC (3/2/5 vol.)**	3.5–4.9 V	65 mA g^{-1} (~C/2) at 25 °C	84% after 200 cycles	3
**1.2 M $LiPF_6$ in EC/EMC (3/7 vol.)**	3.5–4.9 V	1 C at R.T.	92% after 250 cycles	4
**1 M $LiPF_6$ in EC/DMC (1/1 vol.)**	3.5–5.0 V	75 mA g^{-1} (~C/2) at 40 °C	Dead (~10 mAh g^{-1}) at 25 cycles	5
**1 M LiODFB in EC/DMC (1/1 vol.)**	3.7–4.9 V	1 C at 25 °C	76% at 200 cycles	6
**1 M $LiPF_6$ in EC/DMC/DEC (1/1/1 vol.)**	3.5–5.0 V	1 C at R.T.	82% at 150 cycles	7
**1 M $LiPF_6$ in EC/DEC (3/7 vol.)**	3.0–4.9 V	147 mA g^{-1} (1 C) at 30 °C	89% at 300 cycles	8
**1 M $LiPF_6$ in EC/DEC (1/1 vol.)**	3.0–5.0 V	2 C at 30 °C	53% at 300 cycles	9
**1 M $LiPF_6$ in EC/DMC (1/1 vol.)**	3.5–4.9 V	1 C at 25 °C	13% at 300 cycles (33% at 75 cycles)	10
**1 M $LiPF_6$ in EC/DMC (1/2 vol.)**	3.5–4.9 V	1 C at 50 °C	42% at 100 cycles	11

**1 M $LiPF_6$ in PC**	3.5–5.0 V	C/10 (30–40 μA cm^{-2})	90% at 50 cycles	12
**1 M $LiPF_6$ in EC/EMC/DMC (1/1/1 vol.)**	3.5–5.2 V	2 C at 25 ºC	85% at 300 cycles	13
**1.2 M $LiPF_6$ in EC/DMC (3/7 vol.)**	3.5–4.9 V	1 C at 25 ºC	79% at 400 cycles	14
**1 M $LiPF_6$ in EC/DMC (1/3 vol.)**	3.5–4.9 V	C/2 at R.T.	59% at 200 cycles	15

Supplementary Table 3. Performance comparison of Li||Cu cells in conventional mixed-solvent and additive containing carbonate-based electrolytes.

Electrolytes	Current density	Capacity	Cycling performance	Ref.
**1 M $LiPF_6$ in DMDOHD/FEC (5/1 vol.)**	0.5 mA cm^{-2}	1 mAh cm^{-2}	CE ~98% > 200 cycles	This work
**1 M $LiPF_6$ in EC/DMC (1/1 vol.)**	1 mA cm^{-2}	1 mAh cm^{-2}	CE ~85% 70 cycles	16
**1 M $LiPF_6$ in EC/DEC**	1 mA cm^{-2}	1 mAh cm^{-2}	CE ~88% quickly fails within 20 cycles	17
**1 M $LiPF_6$ in EC/EMC**	0.5 mA cm^{-2}	0.5 mAh cm^{-2}	CE ~62.7% Dead at 170 cycles	18
**1 M $LiPF_6$ in FEC/EMC**	0.5 mA cm^{-2}	0.5 mAh cm^{-2}	CE ~83.4% Dead at ~300 cycles	
**1 M $LiPF_6$ in FEC/DMC (1/4 vol.)**	1 mA cm^{-2}	1 mAh cm^{-2}	CE ~33% Dead at ~41 cycles	19
**1 M $LiPF_6$ in EC/DEC (1/1 vol.)**	0.25 mA cm^{-2}	1 mAh cm^{-2}	CE < 80% after 50 cycles	20
**1 M $LiPF_6$ in EC/DEC (1/1 vol.)**	1 mA cm^{-2}	2 mAh cm^{-2}	CE < 10% within 30 cycles	21
**1 M $LiPF_6$ in EC/DEC (1/1 vol.) + 2 wt.% FEC**	1 mA cm^{-2}	2 mAh cm^{-2}	CE ~75% within 30 cycles	22
**1 M $LiPF_6$ in EC/DEC/EMC (1/1/1 vol.)**	1 mA cm^{-2}	1 mAh cm^{-2}	CE < 80% Dead at ~90 cycles	23
**1 M $LiPF_6$ in EC/EMC (1/1 vol.) + 2 wt.% FEC**	0.5 mA cm^{-2}	1 mAh cm^{-2}	CE ~90% Dead at 56 cycles	24

**1 M $LiPF_6$ in EC/EMC (3:7 by wt.) + 2 wt.% VC**	0.5 mA cm^{-2}	1 mAh cm^{-2}	CE ~44% within 40 cycles	25
**1.2 M $LiPF_6$ in EC/EMC (3/7 vol.)**	1 mA cm^{-2}	2 mAh cm^{-2}	CE ~60% Dead at 15 cycles	26
**1.2 M $LiPF_6$ in FEC/EMC (3/7 vol.)**	1 mA cm^{-2}	2 mAh cm^{-2}	CE ~94% Within 20 cycles	
**1.2 M $LiPF_6$ in DFEC/EMC (3/7 vol.)**	1 mA cm^{-2}	2 mAh cm^{-2}	CE ~96% Within 20 cycles	
**1 M $LiPF_6$ in EC/DMC (1/1 vol.)**	0.5 mA cm^{-2}	0.5 mAh cm^{-2}	CE ~84.4% Dead at 20 cycles	27
**1 M $LiPF_6$ in EC/DMC (1/1 vol.) + 5% FEC**	0.5 mA cm^{-2}	0.5 mAh cm^{-2}	CE ~94.1% Dead at 120 cycles	
**1 M $LiPF_6$ in FEC/EC/DMC (2/9/9 vol.)**	1 mA cm^{-2}	1 mAh cm^{-2}	CE ~80% Within 150 cycles	28

Supplementary Table 4. Performance comparison of Li||$LiNi_{0.5}Mn_{1.5}O_4$ cells in conventional mixed-solvent and additive containing carbonate-based electrolytes.

Electrolytes	Voltage (V vs. Li/Li^+)	Cycling condition	Capacity retention	Ref.
**1 M $LiPF_6$ in DMDOHD/FEC (5/1 vol.)**	3.0–4.9 V	C/3 at R.T.	~97% after 250 cycles	This work
**1.3 M $LiPF_6$ in EC/EMC/DEC + 0.2 wt.% TMSPO**	3.5–4.9 V	65 mA g^{-1} (~C/2) at 25 °C	92% after 200 cycles	3
**1.2 M $LiPF_6$ in EC/EMC/F-EPE (3/6/1 vol.)**	3.5–4.9 V	1 C at R.T.	96% after 250 cycles	4
**1 M $LiPF_6$ in EC/DMC (1/1 vol.) + 1 wt.% ADM**	3.5–5.0 V	75 mA g^{-1} (~C/2) at 40 °C	96% at 25 cycles	5
**1 M LiODFB in EC/DMC (1/1 vol.) + 4 wt.% LiDFB**	3.7–4.9 V	1 C at 25 °C	89% at 200 cycles	6
**1 M $LiPF_6$ in EC/DMC/DEC (1/1/1 vol.) + 2 wt.% FEC**	3.5–5.0 V	1 C at R.T.	96% at 150 cycles	7
**1 M $LiPF_6$ in EC/DEC (3/7 vol.) + 10 wt.% ETFEC**	3.0–4.9 V	147 mA g^{-1} (1 C) at 30 °C	91% at 300 cycles	8
**1 M $LiPF_6$ in EC/DEC (1/1 vol.) + 0.2 wt.% PFPDPP**	3.0–5.0 V	2 C at 30 °C	71% at 300 cycles	9
**1 M $LiPF_6$ in EC/DMC (1/1 vol.) + 0.5 wt.% PA or BA or IA**	3.5–4.9 V	1 C at 25 °C	76% or 85% or 66% at 300 cycles	10
**1 M $LiPF_6$ in EC/DMC (1/2 vol.) + 0.5 wt.% DMPP**	3.5–4.9 V	1 C at 50 °C	91% at 100 cycles	11
**1 M $LiPF_6$ in EC/EMC/DMC (1/1/1 vol.) + 0.0025 wt.% PY**	3.5–5.2 V	2 C at 25 °C	93% at 300 cycles	13

**1.2 M $LiPF_6$ in EC/DMC (3/7 vol.) + 0.1 wt.% MPS**	3.5–4.9 V	1 C at 25 ^{o}C	89% at 400 cycles	14

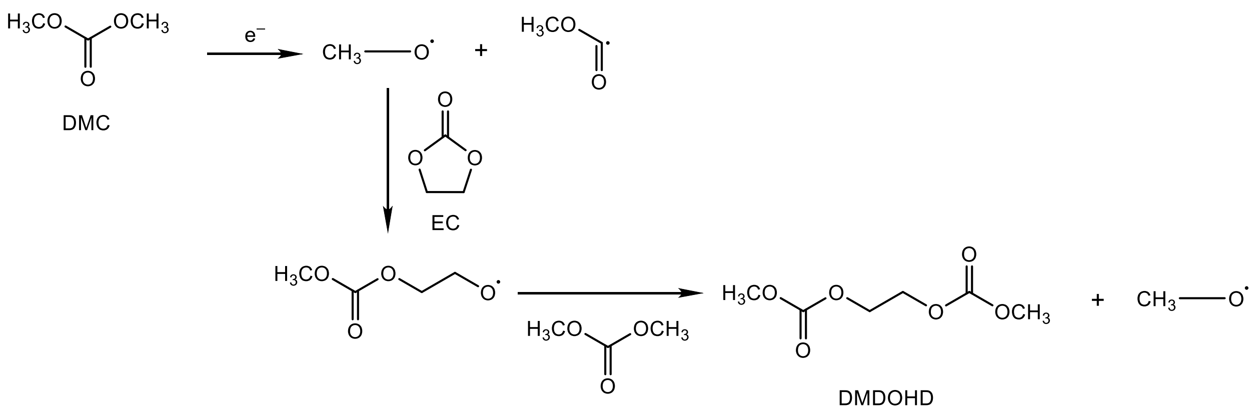

Supplementary Fig. 1 Proposed mechanism for DMDOHD generation in EC/DMC-based electrolytes.

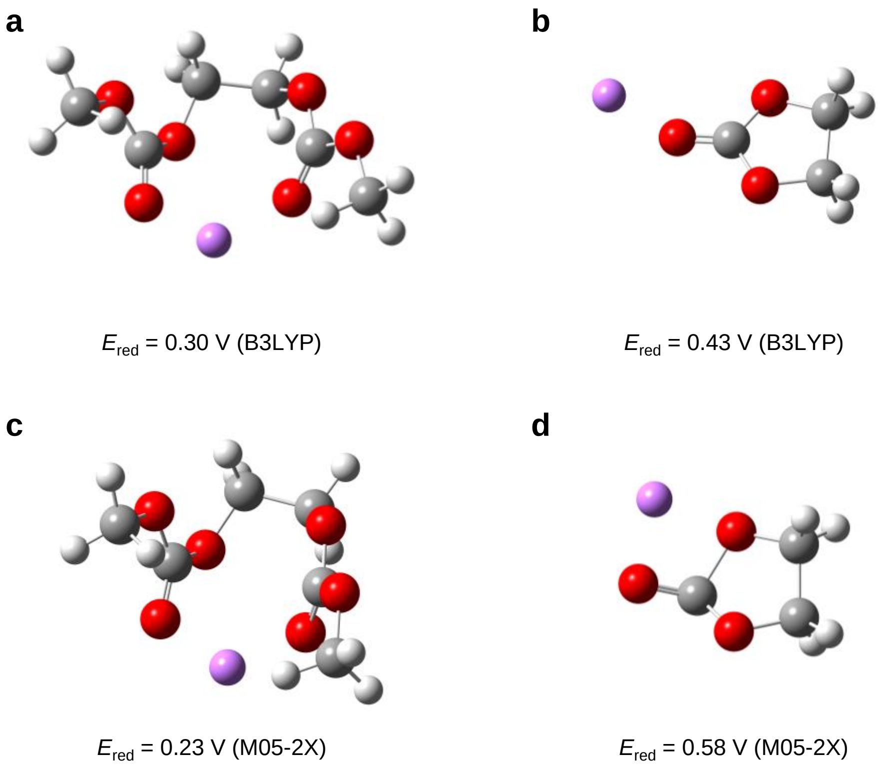

Supplementary Fig. 2 Optimized geometry and reduction potential (vs. Li/Li^+) of (a, c) $DMDOHD/Li^+$ and (b, d) EC/Li^+ complexes attained from (a, b) B3LYP/6-311+G(d, p) and (c, d) M05-2X/6-31+G(d,p) for structure optimization and M05-2X/6-31G(d) for solvation energy calculation with SMD (acetone). Atom colors: Li, purple; C, dark grey; O, red; H, white.

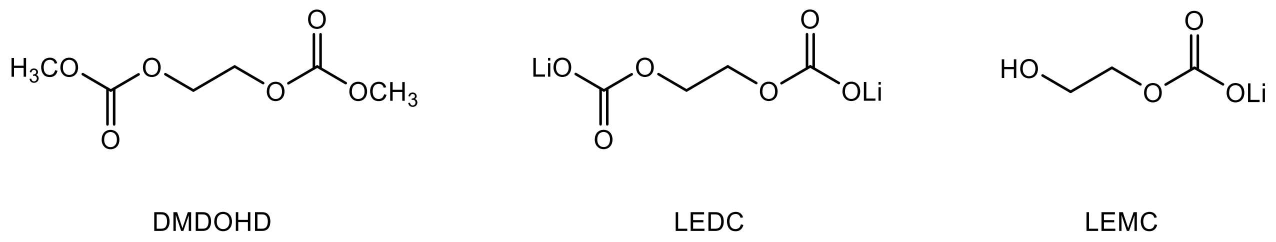

Supplementary Fig. 3 Structural formulas of dimethyl 2,5-dioxahexanedioate solvent (DMDOHD) and two key components of the SEI — dilithium ethylene di-carbonate (LEDC), and lithium ethylene mono-carbonate (LEMC).

Supplementary Note 1

The state-of-the-art electrolytes used in commercial LIBs consist of low concentration lithium salts dissolved in mixed solvents of linear and cyclic carbonates, such as 1 M $LiPF_6$ in ethylene carbonate/dimethyl carbonate (EC/DMC). In general, the linear carbonates possess low viscosities and low melting points (Supplementary Table 6), which enables for the associated electrolytes to have high fluidity (low viscosity), high lithium-ion transport, and a wide service temperature range. However, the linear carbonates are unsuitable as sole solvent because of their incapability to form a stable SEI on the negative electrode surface as well as the limited oxidation (anodic) stability. By contrast, the cyclic carbonates can participate in the formation of an effective SEI by generating a series of lithium alkyl carbonates on the negative electrode surface (e.g., graphitic carbon) through ring-opening reactions and presumably a similar surface passivation reaction pathway of the transitional metal oxide positive electrodes. Nevertheless, their high melting point makes the only available as solvents in a narrow range of temperatures. For example, although EC is a good SEI-forming agent, it cannot be used as an independent solvent at room temperature due to its high melting point (~36.4 ^{o}C). It is also generally difficult to replace EC with other low-melting-point solvents, such as propylene carbonate (PC), for the strict requirement of SEI-forming ability. In addition, their high viscosity also reduces the lithium-ion transport performances.

Interestingly, by using mixtures of linear and cyclic carbonates as solvents, a balance between melting point, viscosity and favorable interfacial chemistry can be achieved. This is the reason why commercial LIBs use electrolyte formulations with lithium salts dissolved in mixed solvents of linear and cyclic carbonates. Nonetheless, there are still some disadvantages existing in the commercial electrolytes based on this formulation: (1) High volatility and flammability. Because of the low boiling and flash points of the linear carbonate components, the commercial carbonate-based electrolytes exhibit high volatility and inherent flammability, which may cause safety issues during battery abuse operations. (2) Low tolerance voltage. At operating voltages above 4.5 V, the carbonate-based electrolytes are anodically unstable and decompose to release CO_2 and O_2, which limits their application to a 5 V-class positive electrode chemistry (e.g., $LiNi_{0.5}Mn_{1.5}O_4$ (LNMO), $LiCoPO_4$ (LCP), etc.). (3) Low lithium plating/stripping CE. Due to the inherent reactivity of the carbonyl functional group towards lithium metal, the commercial carbonate-based electrolytes generally show low lithium plating/stripping CE (< 80% without additives) and uncontrollable lithium dendrite growth.

Supplementary Table 5. Chemical structure and molecular orbital energy of conventional (DEC, EMC, DMC, and EC) as well as of DMDOHD carbonate organic solvents used in battery electrolytes.

Molecule	Structure	Model chemistry	LUMO (eV)	HOMO (eV)
DEC	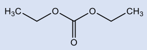	IEFPCM/B3LYP/6-311+G(d,p)	0.20	–8.25
		SMD/B3LYP/6-311+G(d,p)	0.30	–8.02
		SMD/M05-2X/6-311+G(d,p)	1.46	–10.14
		SMD/wB97XD/6-311+G(d,p)	2.13	–10.28
		SMD/G4MP2/6-311+G(d,p)	1.32	–12.43
EMC	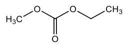	IEFPCM/B3LYP/6-311+G(d,p)	0.18	–8.31
		SMD/B3LYP/6-311+G(d,p)	0.27	–8.08
		SMD/M05-2X/6-311+G(d,p)	1.44	–10.20
		SMD/wB97XD/6-311+G(d,p)	2.09	–10.34
		SMD/G4MP2/6-311+G(d,p)	1.29	–12.56
DMC	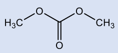	IEFPCM/B3LYP/6-311+G(d,p)	0.15	–8.37
		SMD/B3LYP/6-311+G(d,p)	0.24	–8.14
		SMD/M05-2X/6-311+G(d,p)	1.43	–10.25
		SMD/wB97XD/6-311+G(d,p)	1.96	–10.51
		SMD/G4MP2/6-311+G(d,p)	1.28	–12.80
EC	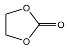	IEFPCM/B3LYP/6-311+G(d,p)	–0.03	–8.63
		SMD/B3LYP/6-311+G(d,p)	0.09	–8.65
		SMD/M05-2X/6-311+G(d,p)	1.32	–10.59
		SMD/wB97XD/6-311+G(d,p)	1.92	–10.83
		SMD/G4MP2/6-311+G(d,p)	1.47	–12.79
DMDOHD	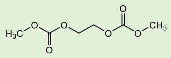	IEFPCM/B3LYP/6-311+G(d,p)	0.06	–8.47
		SMD/B3LYP/6-311+G(d,p)	0.15	–8.25
		SMD/M05-2X/6-311+G(d,p)	1.31	–10.37
		SMD/wB97XD/6-311+G(d,p)	1.96	–10.51
		SMD/G4MP2/6-311+G(d,p)	1.16	–12.73

Supplementary Note 2

For the calculation of the LUMO and HOMO energy levels, the selection of the implicit solvent model was determined based on the dielectric constants of the studied carbonate molecules. For linear carbonate molecules such as DMC (ε=3.12), EMC (ε=2.93), DEC (ε=2.82), and DMDOHD (ε=2.9, calculated value), their dielectric constants exhibit minimal variation and are closely aligned (around 3), thus we use ethyl acetate, which has a similar molecule structure and a close dielectric constant (ε=5.99), as the implicit solvent model. While the cyclic carbonate molecule EC possesses a significantly higher dielectric constant of 89.6, prompting the adoption of water as an implicit solvent model, given its similarly large dielectric constant of 78.5. Additionally, we also used EC with known dielectric constant (ε=89.6) as the research object to explore the effect of different implicit solvent models with varying dielectric constants, such as diethylether (ε=4.22), acetone (ε=20.5), methanol (ε=33.7), acetonitrile (ε=37.5), and water (ε=78.5), on the results of HOMO and LUMO energy level calculations. We found that the implicit solvent models with different dielectric constants had minimal impact on the LUMO calculation results of EC (diethylether: –8.36 eV, acetone: –8.38 eV, methanol: –8.66 eV, acetonitrile: –8.41 eV, water: –8.65 eV). However, they did significantly affect the HOMO calculation results, with implicit solvents possessing higher dielectric constants (except for methanol) leading to more negative calculated HOMO results for EC (diethylether: 0.08 eV, acetone: 0.09 eV, methanol: 0.09 eV, acetonitrile: 0.09 eV, water: 0.09 eV). This underscores the importance of selecting an appropriate implicit solvent model. In general, it is advisable to choose solvents with similar structures and close dielectric constants as implicit solvent models when calculating LUMO and HOMO energy levels. Interestingly, it is worth mentioning that the HOMO energy levels of the EC calculated using the five different implicit solvent models mentioned above are all lower than those of the other studied carbonate molecules (DEC: –8.02 eV, EMC: –8.08 eV, DMC: –8.14 eV, and DMDOHD: –8.25 eV) calculated by the same model chemistry (SMD/B3LYP/6-311+G(d,p)), which does not affect the results of the comparison of the energy level trends.

It’s worth noting that different functional methods have their unique strengths and weaknesses for calculating molecular orbitals. It’s better to use different functional methods to compare the trends in LUMO and HOMO energy levels for the studied carbonate molecules more comprehensively. In Supplementary Table 5, we provide a summary of the LUMO and HOMO energy levels calculated for DEC, EMC, DMC, EC, and DMDOHD using various functional methods. Below are the orders of LUMO and HOMO energy levels for the five carbonate molecules calculated with various functional methods:

LUMO energy order:

IEFPCM/B3LYP: DEC>EMC>DMC>DMDOHD>EC

SMD/B3LYP: DEC>EMC>DMC>DMDOHD>EC

SMD/M05-2X: DEC>EMC>DMC>EC>DMDOHD

SMD/wB97XD: DEC>EMC>DMC=DMDOHD>EC

SMD/G4MP2: EC>DEC>EMC>DMC>DMDOHD

HOMO energy order:

IEFPCM/B3LYP: DEC>EMC>DMC>DMDOHD>EC

SMD/B3LYP: DEC>EMC>DMC>DMDOHD>EC

SMD/M05-2X: DEC>EMC>DMC>DMDOHD>EC

SMD/wB97XD: DEC>EMC>DMC=DMDOHD>EC

SMD/G4MP2: DEC>EMC>DMDOHD>EC>DMC

It’s important to note that the LUMO and HOMO energy levels calculated by SMD/G4MP2 appear to contradict the conventional belief that EC should be reduced before DMC. According to the conventional belief, the LUMO energy level of EC should be lower than that of DMC. Additionally, it has been demonstrated that the oxidation potential of high-purity EC should be higher than that of high-purity DMC,[29] suggesting that the HOMO energy level of EC should also be lower than that of DMC. However, the results attained from SMD/G4MP2 indicate that both the LUMO and HOMO energy levels of EC is higher than that of DMC.

Except for SMD/G4MP2, the energy level trends from other four functional methods largely support our conclusions in main text — "(1) For the SEI film forming ability, our theoretical calculation results show that the energy of the lowest unoccupied molecular orbital (LUMO) in DMDOHD is lower than that of conventional linear carbonates, (e.g., diethyl carbonate - DMC, ethyl methyl carbonate – EMC, or diethyl carbonate - DEC), and close to that of ethylene carbonate - EC (Supplementary Table 5). This indicates that DMDOHD is preferentially reduced to form the SEI under cathodic polarization with an essential role to play in the modulation of the SEI composition and interfacial chemistry, like EC. And (2) For the anodic stability, DMDOHD also exhibits a lower than DMC, EMC and DEC highest occupied molecular orbital (HOMO) energy level (Supplementary Table 5), implying improved anodic stability of the DMDOHD solvent."

Supplementary Table 6. The oxidation potential of DMDOHD, PF_6^- and DMDOHD/PF_6^- complex calculated using the Nernst equation.

Species	Oxidation potential (vs. Li/Li^+)
DMDOHD/PF_6^-	5.05
DMDOHD	6.40
PF_6^-	8.97

Supplementary Note 3

Prior studies reveal that the composition of the CEI is primarily governed by the prevalence of species within the inner Helmholtz layer.[30] Typically, during the charging phase (i.e., when the voltage rises), anions such as PF_6^- and solvent molecules with high dielectric constant (i.e., high polarity) such as EC, tend to accumulate on the positive electrode surface propelled by elevated electric field.[31] Research by Borodin and Xu *et al.* indicated that, at low salt concentrations, only approximately 30% of the inner Helmholtz layer is occupied by anions.[32,33] Consequently, for the 1 M $LiPF_6$/EC-DMC system, the species on the positive electrode surface primarily include free EC solvent molecules, free PF_6^- anions, and EC/PF_6^- complexes. Numerous investigations establish that the anodic stability of free PF_6^- anion and free EC solvent molecule exceeds 6 V (vs. Li/Li^+).[32,34–36] However, the coordination of EC with PF_6^- anions to form an EC/PF_6^- complex significantly reduces the oxidation potential.[32,34–36] Additionally, when EC undergoes dehydrogenation and decomposition into CO_2 and OC_2H_3 through ring opening, the oxidation potential decreases to approximately 4.5 V (vs. Li/Li^+).[32,34–36] Hence, for the 1 M $LiPF_6$/EC-DMC system, the CEI primarily results from the ring-opening dehydrogenation decomposition of free EC molecules and the oxidation of the EC/PF_6^- complex, encompassing the decomposition of a majority of EC and a minor fraction of PF_6^-.[30,31] The constituents of the resulting CEI predominantly comprise organic species generated through the decomposition of EC.[30,31] However, organic SEI is typically characterized as less dense (thick and porous) and less robust.[30,37] This characteristic not only fails to effectively suppress the irreversible oxidation of the electrolyte under high voltage but also impedes the transport of lithium ions, posing challenges to the stable cycling of high-voltage positive electrodes.[30,37]

In contrast to EC (ε=89.6), DMDOHD (ε=2.9) will exhibit minimal accumulation on the positive electrode surface during charging due to its significantly lower dielectric constant. Consequently, primarily PF_6^- anions accumulate on the positive electrode surface. However, research by Borodin and Xu *et al.* further suggested that PF_6^- anions can only partially replace solvent molecules in the inner Helmholtz layer,[32,33] leaving some DMDOHD solvent molecules near the positive electrode surface. As previously

discussed, the oxidation potential of free PF_6^- anions and free solvent molecules are generally higher, while solvent/PF_6^- complexes have lower oxidation potentials.[32,34–36] This observation is also supported by our calculation results, as indicated in Supplementary Table 6. The oxidation stability of free DMDOHD and PF_6^- anions is higher than 6 V (vs. Li/Li$^+$), specifically 6.40 and 8.97 V (vs. Li/Li$^+$), respectively, while the oxidation potential of the DMDOHD/PF_6^- complex is significantly reduced to 5.05 V. In fact, numerous experiments and theoretical calculations consistently reveal that the theoretically calculated oxidation potential of the electrolyte tends to be higher than that observed in practical experiments. Moreover, most of the high-voltage transition metal positive electrode materials may also have a catalytic effect on the decomposition of the electrolyte in the actual battery system, resulting in an earlier oxidation potential of the electrolyte. Therefore, we believe that the actual oxidation potential of DMDOHD/PF_6^- complex should be lower than 5 V (vs. Li/Li$^+$). Thus, in the 1 M $LiPF_6$/DMDOHD system, the CEI will be formed predominantly through anodic decomposition of the DMDOHD/PF_6^- complex. In comparison with the 1 M $LiPF_6$/EC-DMC system, the CEI in the 1 M $LiPF_6$/DMDOHD system will feature a lower oxidative decomposition contribution from free DMDOHD solvent molecules. Consequently, the content of inorganic species and fluorine species in the CEI formed by the 1 M $LiPF_6$/DMDOHD system will be higher than that in the 1 M $LiPF_6$/EC-DMC system, thus promoting the formation of a denser, more uniform, and robust CEI, which is able to effectively suppress further electrolyte decomposition and maintain the stability of the positive electrode/electrolyte interface.[30,37–39] Based on these analysis, we believe that DMDOHD with low dielectric constant has the ability to participate in CEI film formation and to increase the content of inorganic species and fluorine species to form a dense, uniform and robust CEI, thereby endowing the positive electrode material with the ability to cycle stably over an extended period.[30,37–39]

Supplementary Table 7. The physical properties of cyclic and linear organic carbonates.

	Solvent	Structure	M.wt	T_m/°C	T_b/°C	T_f/°C	η/mPa s at 25 °C	ε at 25 °C	D/g cm^{-3} at 25 °C
Cyclic	EC	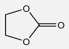	88	36.4	248	160	1.9 (40 °C)	89.78	1.32
	PC	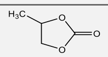	102	-48.8	242	132	2.53	64.92	1.20
	FEC	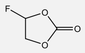	106	18~23	212	128	4.1 (40 °C)	78.40	1.48
	VC	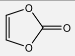	86	22	178	73	-	-	1.35
Linear	DMC	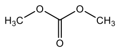	90	4.6	91	18	0.59 (20 °C)	3.10	1.06
	DEC	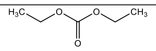	118	-43	126	31	0.75	2.81	0.97
	EMC	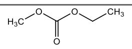	104	-14.5	107	23	0.65	2.96	1.01
	DMDOHD	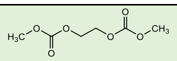	178	-	220	82*	7.582[#]	2.9[&]	1.25[Δ]

Note: The values in this table were retrieved from literature (refer to *Chem. Rev.*, **2004**, *104*, 4303) and databases.

*https://starshinechemical.com/isomer/dimethyl-25-dioxahexanedioate/;

[#]Experimental data shown in Supplementary Table 9.

[&]Computed value.

[Δ]Experimental value.

Supplementary Table 8. The ionic conductivity and activation energy of ion transport in 1 M-LPF-DMC, 1 M-LPF-EC/DMC and 1 M-LPF-DMDOHD electrolytes at different temperatures estimated from the data presented in Fig. 2a.

Electrolyte	σ at 20 °C ($mS\ cm^{-1}$)	σ at 40 °C ($mS\ cm^{-1}$)	σ at 70 °C ($mS\ cm^{-1}$)	E_a (eV)
1 M-LPF-DMC	2.6	3.1	4.7	0.06
1 M-LPF-EC/DMC	7.8	10.5	16.2	0.14
1 M-LPF-DMDOHD	0.2	0.6	1.8	0.38

Supplementary Table 9. Electrolyte viscosity at 25 °C.

Electrolyte solvents or electrolyte formulation	Viscosity at 25 °C (mPa s)
DMC	0.59 (20 ºC)
EC/DMC	1.68
DMDOHD	7.58
1 M-LPF-DMC	1.75
1 M-LPF-EC/DMC	3.92
1 M-LPF-DMDOHD	62.97
1 M-LPF-DMDOHD/FEC (5/1)	29.73

Note: Viscosity value for DMC was sourced from *Chem. Rev.*, **2004**, *104*, 4303, while viscosity values for other solvents and electrolytes were attained from our experimental tests.

Supplementary Table 10. Comparison of the ionic conductivity of the 1 M-LPF-DMDOHD electrolyte with ionic liquids, solid ceramic and polymer electrolytes used in LMBs or LIBs.

Electrolytes		Ionic conductivity (mS cm^{-1})	Ref.
**1 M $LiPF_6$ in DMDOHD**		0.21 (25 °C)	This work
Solid ceramic	$Li_{6.75}La_3Zr_{1.75}Nb_{0.25}O_{12}$	0.092 (25 °C)	40
	$Li_{1.3}Al_{0.3}Ti_{1.7}(PO_4)_3$	0.0778 (30 °C)	41
	$Li_{1+x}Al_xTi_{2-x}(PO_4)_3$	0.052 (25 °C)	42
	$Li_6BaLa_2Ta_2O_{12}$	0.04 (22 °C)	43
	$Li_7La_3Zr_2O_{12}$	0.244 (25 °C)	44
	$Li_{6.28}Al_{0.24}La_3Zr_2O_{11.98}$	0.027 (25 °C)	45
	Ga-$Li_7La_3Zr_2O_{12}$	0.12 (30 °C)	46
	$Li_{0.34(1)}La_{0.51(1)}TiO_{2.94(2)}$	0.02 (25 °C)	47
	LiPON	0.0012 (25 °C)	48
**Ionic liquid**	$[nOctBu_3N]^+[N(CF_3SO_2)_2]^-$	0.13 (25 °C)	49

	$[Bu_3He_xN]^+[N(CF_3SO_2)_2]^-$	0.16 (25 °C)	49
	LiLL-0	0.00530 (25 °C)	
	LiIL-1	0.0439 (25 °C)	
	LiIL-2	0.115 (25 °C)	
	LiIL-3	0.0532 (25 °C)	
	LiIL-4	0.104 (25 °C)	
	LiIL-5	0.0243 (25 °C)	50
	LiIL-6	0.145 (25 °C)	
	LiIL-7	0.187 (25 °C)	
	LiIL-8	0.189 (25 °C)	
	LiIL-9	0.0813 (25 °C)	
	LiIL-10	0.0940 (25 °C)	
**Polymer electrolyte**	PEO+P(STFSILi)	0.013 (60 °C)	51

	PDOL GPE	0.0616 (25 °C)	52
	poly(alkyl fluoroacrylate)	0.102 (25 °C)	53
	pDOL with $Sn(OTf)_2$	0.0616 (25 °C)	52
	PVDA	0.033 (25 °C)	54
	Poly(ethylene glycol) methyl ether acrylate	0.1 (40 °C)	55

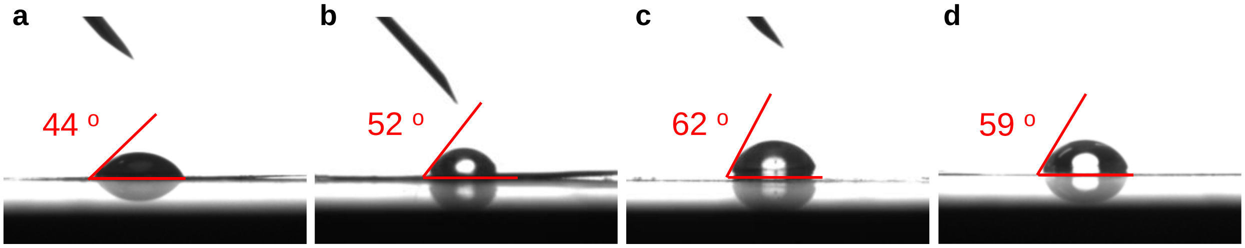

Supplementary Fig. 4 Digital photographs of the static contact angles of (a) 1 M-LPF-DMC, (b) 1 M-LPF-EC/DMC, (c) 1 M-LPF-DMDOHD and (d) 1 M-LPF-DMDOHD/FEC (5/1 vol) electrolytes on the surface of polypropylene (PP) Celgard separator.

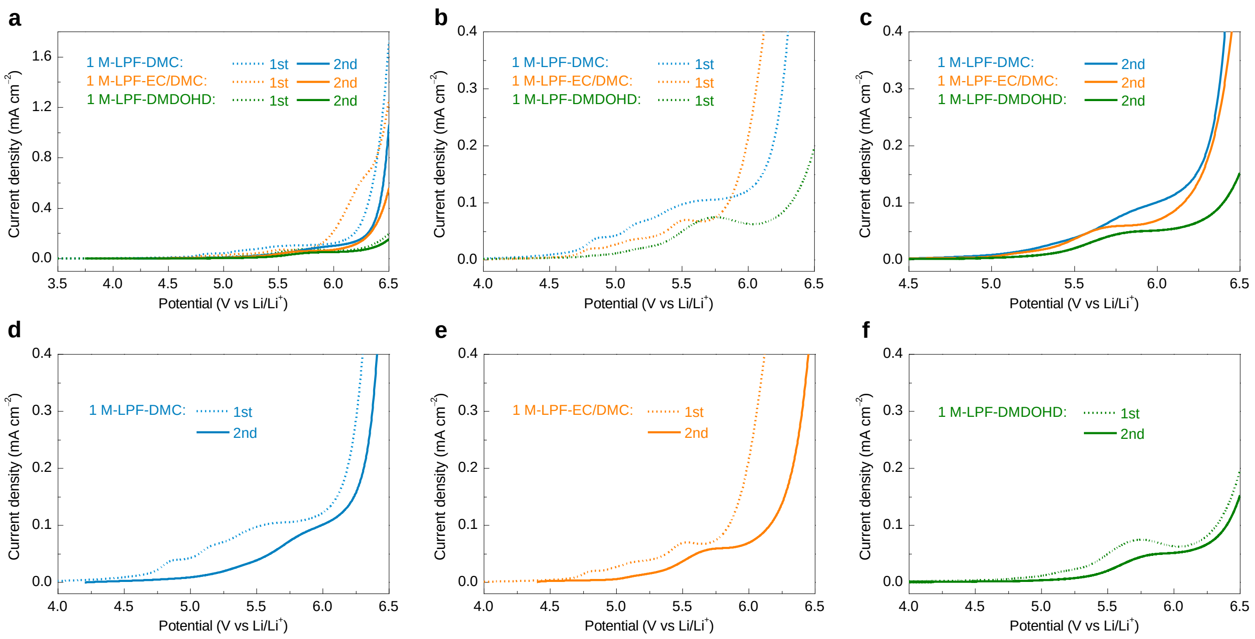

Supplementary Fig. 5 (a) Linear scan voltammetry (LSV) plots of 1 M-LPF-DMC, 1 M-LPF-EC/DMC and 1 M-LPF-DMDOHD electrolytes measured from open-circuit voltage (OCV) to 6.5 V (vs. Li^+/Li). The measurements were conducted by using a three-electrode electrochemical cell with a 3 mm diameter glassy carbon (GC) disk as the working electrode and two lithium metal foils as the counter and reference electrodes, respectively. The scan rate is 1 mV s^{-1}. (b, c) Enlarged LSV plots of (b) the first scan and (c) the second scan of 1 M-LPF-DMC, 1 M-LPF-EC/DMC and 1 M-LPF-DMDOHD electrolytes shown in (a). (d−f) Enlarged LSV plots of (d) 1 M-LPF-DMC, (e) 1 M-LPF-EC/DMC and (f) 1 M-LPF-DMDOHD electrolytes shown in (a).

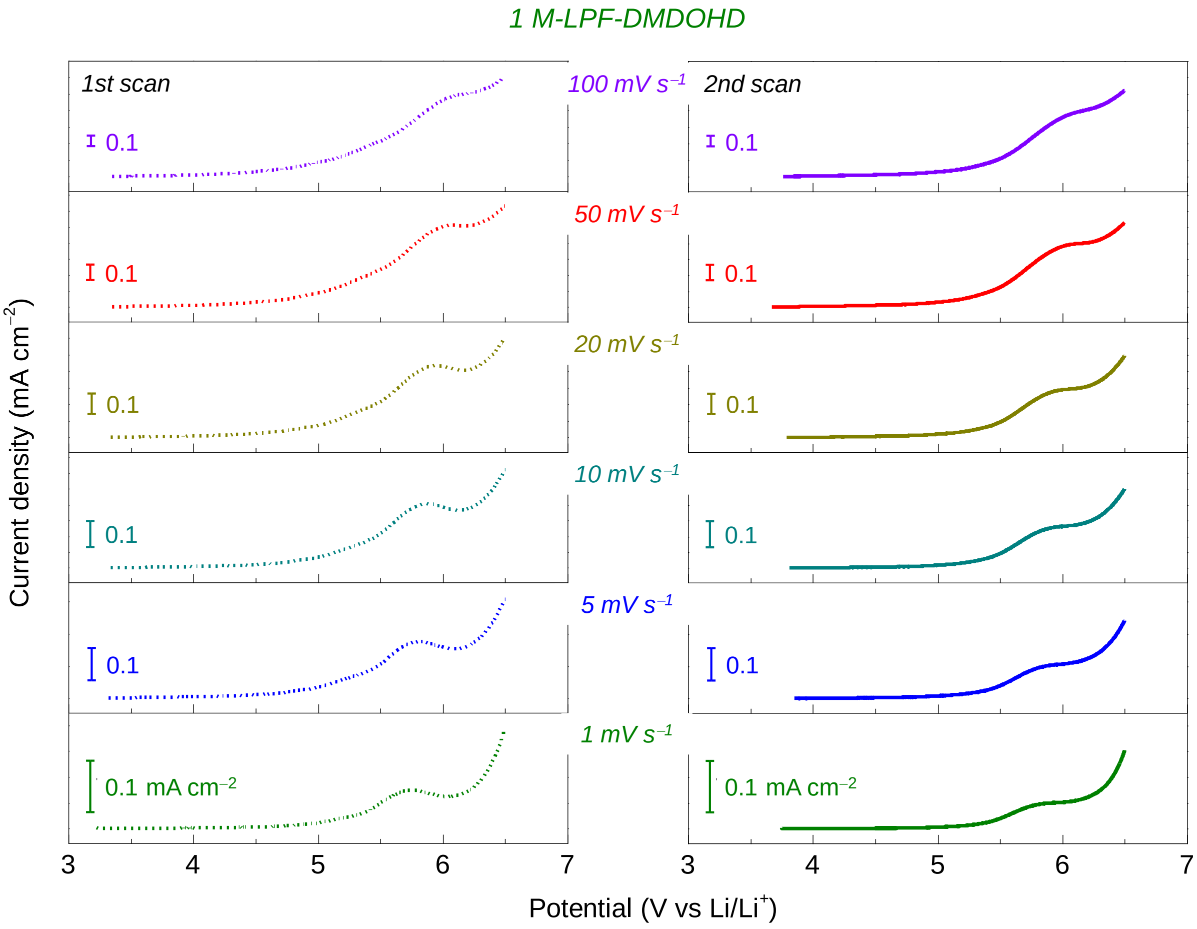

Supplementary Fig. 6 Linear scan voltammetry (LSV) plots of 1 M-LPF-DMDOHD electrolytes measured from open-circuit voltage (OCV) to 6.5 V (vs. Li^+/Li) at scan rates varying from 1 to 100 mV s^{-1}. The measurement was conducted by using a three-electrode electrochemical cell with a 3 mm diameter glassy carbon (GC) disk as the working electrode and two lithium metal foils as the counter and reference electrodes, respectively. The GC electrode was polished, cleaned, and thoroughly dried before each measurement at different scan rates. This step aimed to minimize the impact of the passivation film formed on the GC surface by the electrolyte decomposition in the prior experiment.

Supplementary Note 4

As depicted in Supplementary Fig. 5, during the first anodic scan, the onset oxidation potentials for 1 M-LPF-DMC, 1 M-LPF-EC/DMC, and 1 M-LPF-DMDOHD electrolytes were 4.3, 4.6, and 4.8 V (vs. Li^+/Li), respectively. Additionally, the anodic currents when polarized to 6.5 V (vs. Li^+/Li) were 1.7, 1.3, and 0.19 mA cm^{-2}, respectively. However, in the second anodic scan, all three electrolytes exhibited delayed onset oxidation potentials, at 4.8, 5.0, and 5.2 V (vs. Li^+/Li), respectively. Furthermore, the anodic currents at polarization to 6.5 V (vs. Li^+/Li) decreased to 1.1, 0.6, and 0.15 mA cm^{-2}, respectively. This phenomenon suggests that the passivation film formed by the electrolyte decomposition on the glassy carbon (GC) disk electrode surface during the first anodic scan can partially inhibit further electrolyte decomposition. Given that the 1 M-LPF-DMDOHD electrolyte demonstrates higher onset oxidation potentials and lower anodic currents compared to the two carbonate-based reference electrolytes, it can be concluded that the 1 M-LPF-DMDOHD electrolyte offers better anodic stability.

Further, to explore whether the transport limitations caused by the low ionic conductivity may have an effect on the LSV results, we conducted LSV experiments on the 1 M-LPF-DMDOHD electrolyte system at various scan rates, ranging from 1 to 100 mV s^{-1}. Before each measurement at different scan rates, we polished, cleaned and thoroughly dried the GC electrodes to eliminate any influence from the passivation film that might have formed on the GC electrode surface due to electrolyte decomposition in previous experiments. As displayed in Supplementary Fig. 6, our findings reveal that the onset oxidation potentials of the first and second anodic scans of the 1 M-LPF-DMDOHD electrolyte system stabilized at around 4.8 and 5.2 V (vs. Li^+/Li), respectively, while the anodic oxidation current gradually increased as the scan rate was gradually increased from 1 to 100 mV s^{-1}. This phenomenon strongly suggests that the LSV tests on the 1 M-LPF-DMDOHD electrolyte were not affected by mass transport limitations. Moreover, the small anodic oxidation current of the 1 M-LPF-DMDOHD electrolyte can indeed be attributed to its superior anodic stability. These conclusions are bolstered by the following analysis: as the scan rate progressively increased, the reaction rate at the electrode surface accelerated. If the mass transport properties of the 1 M-LPF-DMDOHD electrolyte were inadequate, it would have been increasingly challenging for mass transport to match the escalating reaction rate. In such a scenario, it would have been unlikely to observe the phenomenon in which the anodic oxidation current gradually increased with the rising scan rate.

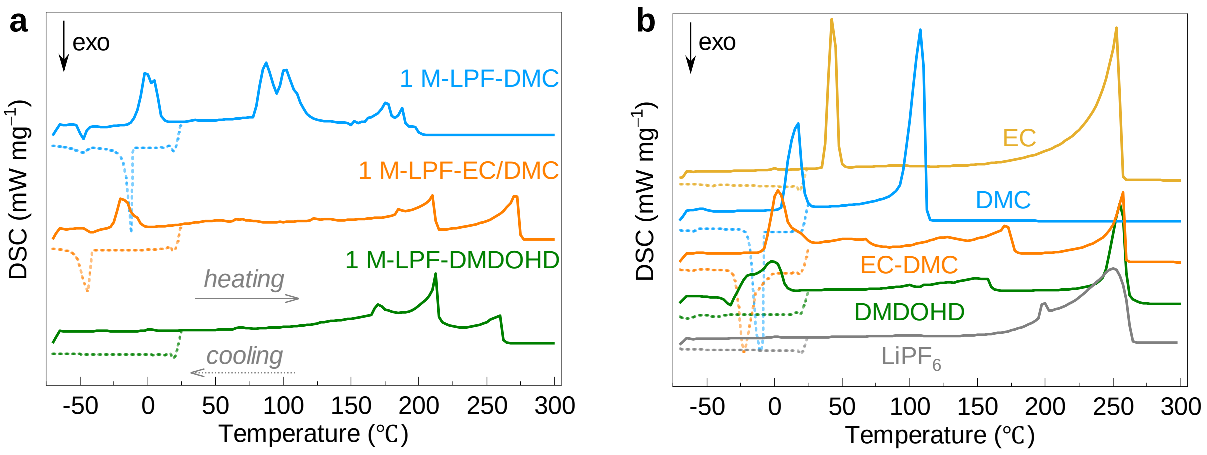

Supplementary Fig. 7 Differential scanning calorimetry curves of (a) three different electrolytes and (b) the corresponding electrolyte solvents and lithium salt.

The thermal stability of the three electrolytes and the corresponding electrolyte solvents and lithium salt were measured on Netzsch DSC 214 differential scanning calorimetry (DSC) analyzer at a heating rate of 10 °C min^{-1} with N_2 as carrier gas from 25 °C to −70 °C and then to 300 °C.

As shown in Supplementary Fig. 7a, in the low temperature region (−70~25 °C), the crystallization/melting temperatures of 1 M-LPF-DMC and 1 M-LPF-EC/DMC electrolytes are −11.9/−1.5 °C and −43.9/−19.3 °C, respectively, whereas the 1 M-LPF-DMDOHD electrolyte does not show any crystallization/melting behavior in this region, indicating that it has a wide liquidus range. In the high temperature region (25~300 °C), for 1 M-LPF-DMC electrolyte, the endothermic peak at 25~126 °C is mainly due to the volatilization of DMC (combined with the blue line in Supplementary Fig. 7b, the boiling point of DMC is of 109.4 °C), while the endothermic peak at 145~205 °C is mainly attributed to the thermal decomposition of $LiPF_6$ (combined with the gray line in Supplementary Fig. 7b, the thermal decomposition range of $LiPF_6$ is 140~268 °C). For 1 M-LPF-EC/DMC electrolyte, the EC/DMC mixed solvent has a thermal reaction between 32 and 180 °C (the endothermic heat is about 102.5 J g^{-1}) and a boiling point of 257.2 °C (see the orange line in Supplementary Fig. 7b). By integrating the DSC curve of

1 M-LPF-EC/DMC electrolyte, we can attain its total endothermic heat in the high temperature region which is as high as 468.7 J g^{-1}. As for the 1 M-LPF-DMDOHD system, the DMDOHD solvent has a weak thermal reaction between 70 and 165 °C (the endothermic heat is only 72 J g^{-1}) and a boiling point of 255.3 °C (see green line in Supplementary Fig. 7b). By integrating its DSC curve, we found its total endothermic heat in the high temperature region is around 445.3 J g^{-1}. Therefore, based on the above analysis, the 1 M-LPF-DMDOHD electrolyte has better thermal stability as compared to 1 M-LPF- DMC and 1 M-LPF-EC/DMC electrolytes, since it has the highest initial thermal reaction temperature and lower total endothermic heat than 1 M-LPF-EC/DMC electrolyte in the high temperature region.

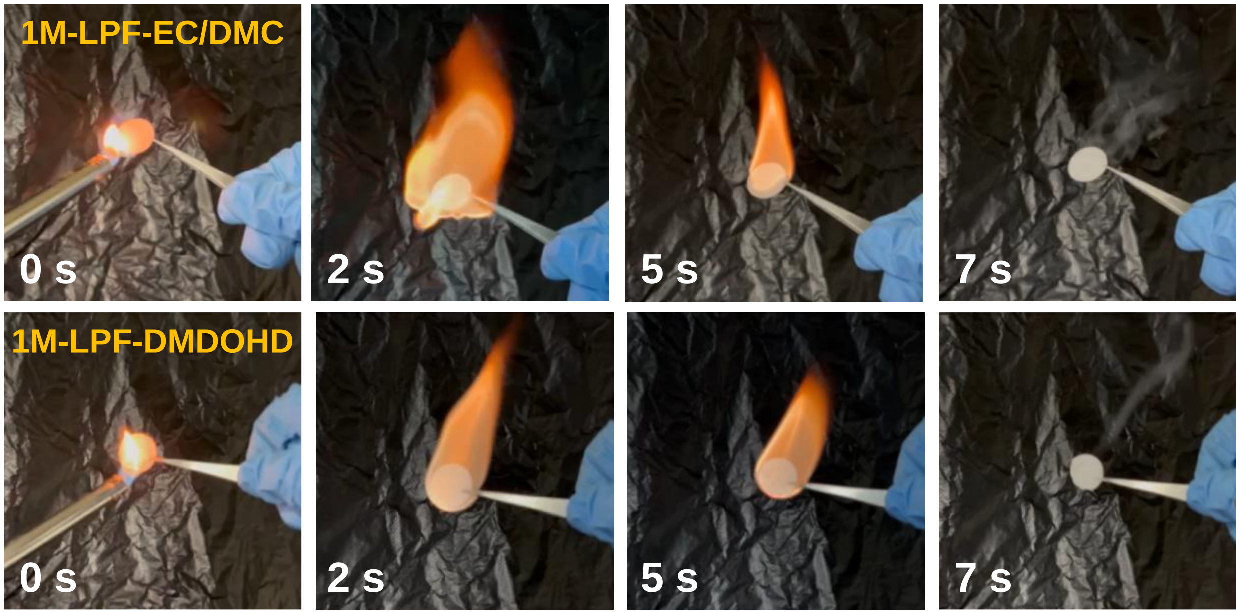

Supplementary Fig. 8 Flammability tests for the conventional 1 M-LPF-EC/DMC and of the 1 M-LPF-DMDOHD electrolytes in the conditions of ignition of a suspended separator impregnated with electrolyte. The detailed parameters of the two electrolytes are shown in Supplementary Table 11. The resulting SET of the conventional 1 M-LPF-EC/DMC and of the 1 M-LPF-DMDOHD electrolytes is 58.8 and 53.8 s g^{-1}, respectively. Refer to Supplementary Videos 5 and 6.

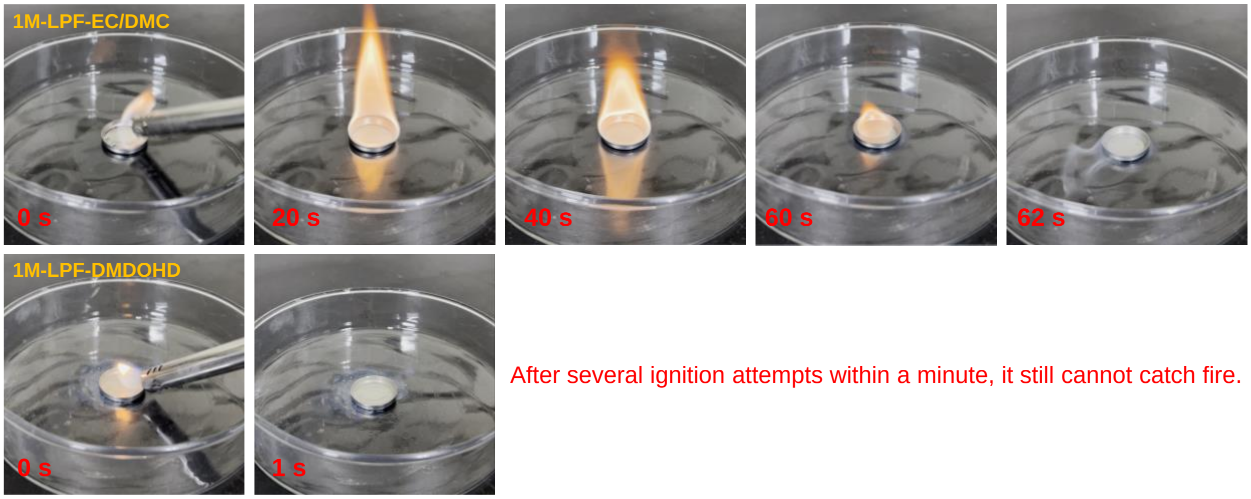

Supplementary Fig. 9 Flammability tests for the conventional 1 M-LPF-EC/DMC and of the 1 M-LPF-DMDOHD electrolytes in the condition of ignition of an electrolyte in a stainless-steel coin cell case. The detailed parameters of the two electrolytes are shown in Supplementary Table 11. The resulting SET of the conventional 1 M-LPF-EC/DMC and of the 1 M-LPF-DMDOHD electrolytes is 85.4 and 0 s g^{-1}, respectively. Refer to Supplementary Videos 7 and 8.

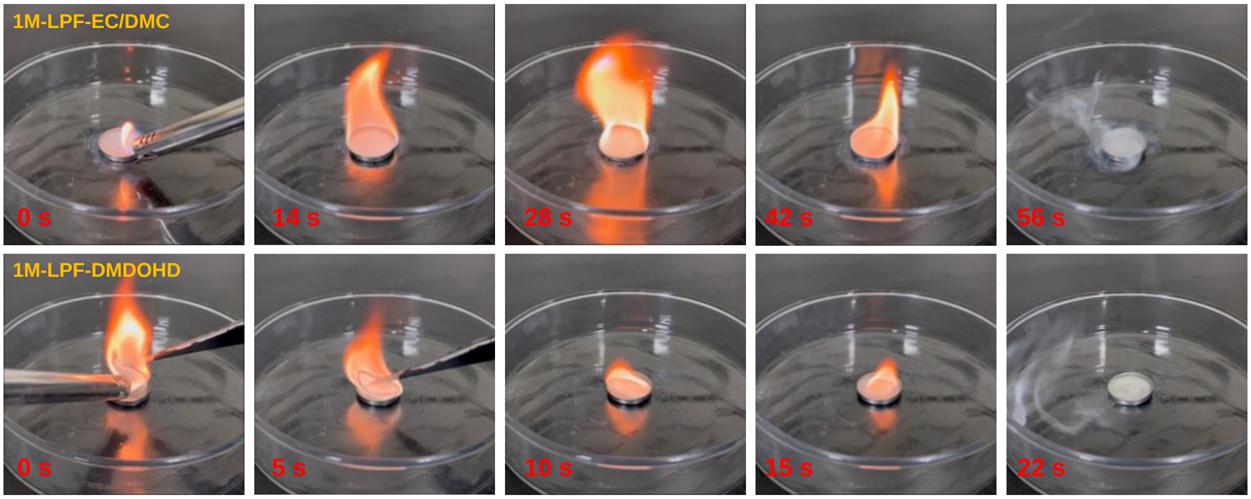

Supplementary Fig. 10 Flammability tests for the conventional 1 M-LPF-EC/DMC and of the 1 M-LPF-DMDOHD electrolytes in the condition of ignition of a separator immersed in electrolyte in a stainless-steel coin cell case. The detailed parameters of the two electrolytes are shown in Supplementary Table 11. The resulting SET of the conventional 1 M-LPF-EC/DMC and of the 1 M-LPF-DMDOHD electrolytes is 79.5 and 29.2 s g^{-1}, respectively. Refer to Supplementary Videos 9 and 10.

Supplementary Table 11. Comparison of the self-extinguishing time (SET) of the 1 M-LPF-DMDOHD and 1 M-LPF-EC/DMC electrolytes under three different conditions.

Conditions	Electrolyte	Volume (μL)	Mass (g)	Burning time (s)	SET (s g^{-1})
**Ignition of a suspended separator impregnated with electrolyte**	1 M-LPF-EC/DMC	100	0.119	7	58.8
	1 M-LPF-DMDOHD	100	0.13	7	53.8
**Ignition of an electrolyte in a stainless-steel coin cell case**	1 M-LPF-EC/DMC	600	0.726	62	85.4
	1 M-LPF-DMDOHD	600	0.754	0	0
**Ignition of a separator immersed in electrolyte in a stainless-steel coin**	1 M-LPF-EC/DMC	600	0.704	56	79.5
	1 M-LPF-DMDOHD	600	0.754	22	29.2

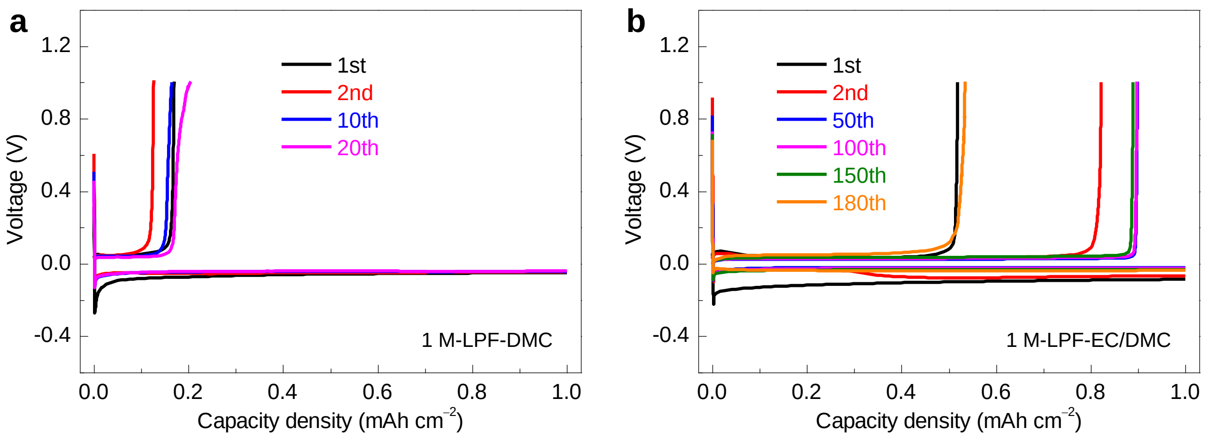

Supplementary Fig. 11 Lithium plating/stripping galvanostatic profiles of Li||Cu cells cycled in (a) 1 M-LPF-DMC and (b) 1 M-LPF-EC/DMC electrolytes at a current density of 0.5 mA cm^{-2} with a cutoff capacity of 1 mAh cm^{-2}.

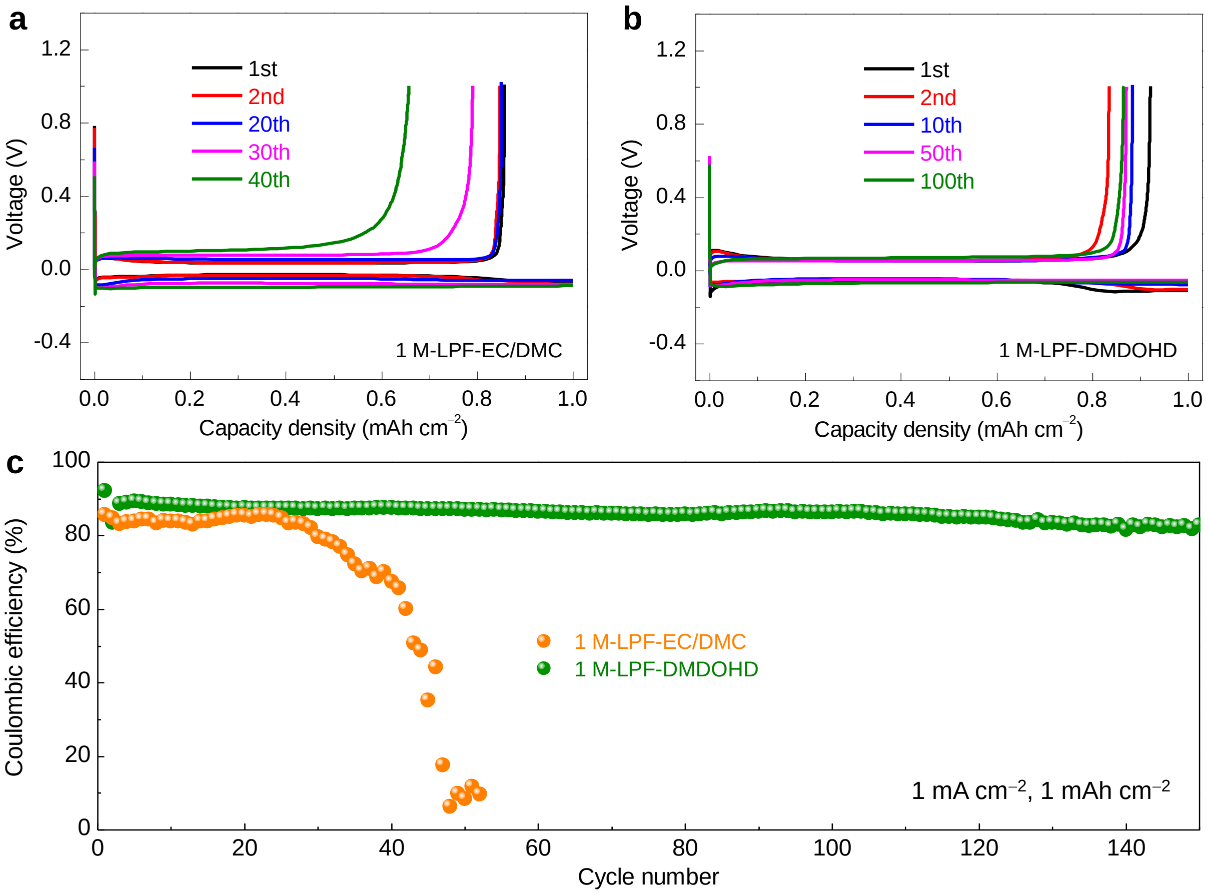

Supplementary Fig. 12 Electrochemical performance comparison of Li||Cu cell with different electrolytes. (a, b) Lithium plating/stripping galvanostatic profile of the Li||Cu cell cycled in (a) 1 M-LPF-EC/DMC and (b) 1 M-LPF-DMDOHD electrolytes at a current density of 1 mA cm^{-2} with a cutoff capacity of 1 mAh cm^{-2}. (c) The associated evolution of the Coulombic Efficiency for the lithium plating/stripping process. The data were recorded after two activation cycles at at a current density of 0.2 mA cm^{-2} and a cutoff capacity of 1 mAh cm^{-2}.

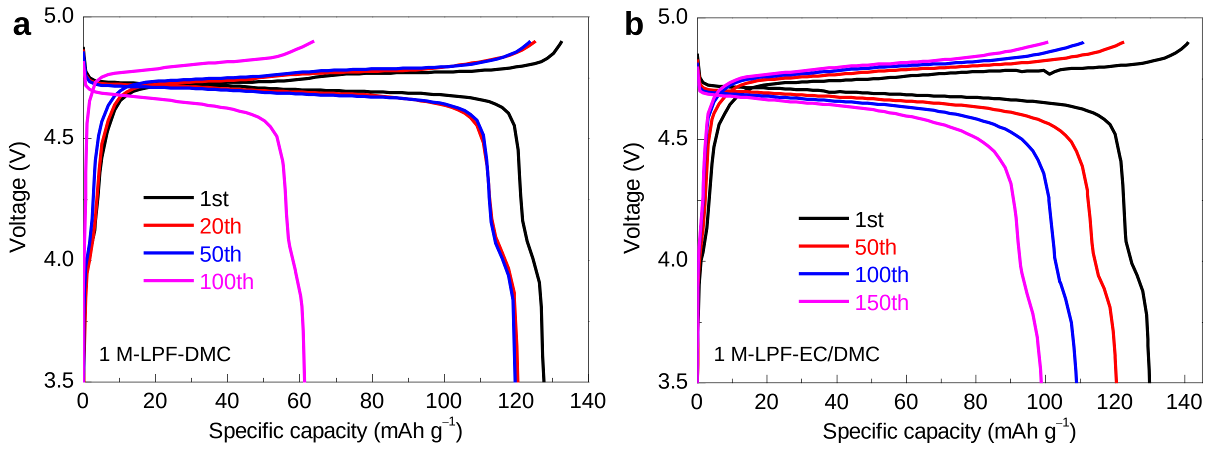

Supplementary Fig. 13 Galvanostatic charge-discharge curves of the Li||$LiNi_{0.5}Mn_{1.5}O_4$ cell cycled in (a) 1 M-LPF-DMC and (b) 1 M-LPF-EC/DMC electrolytes at a C-rate of C/5 (equivalent of a current density of 28.4 mA g^{-1}).

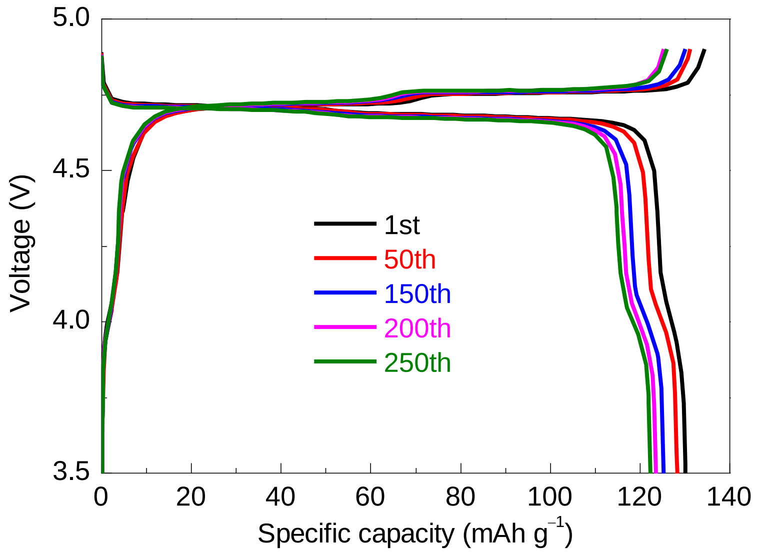

Supplementary Fig. 14 Galvanostatic charge-discharge curves of the Li||$LiNi_{0.5}Mn_{1.5}O_4$ cell cycled in 1 M-LPF-DMDOHD electrolyte at a C-rate of C/3 (equivalent of a current density of 47.3 mA g^{-1}).

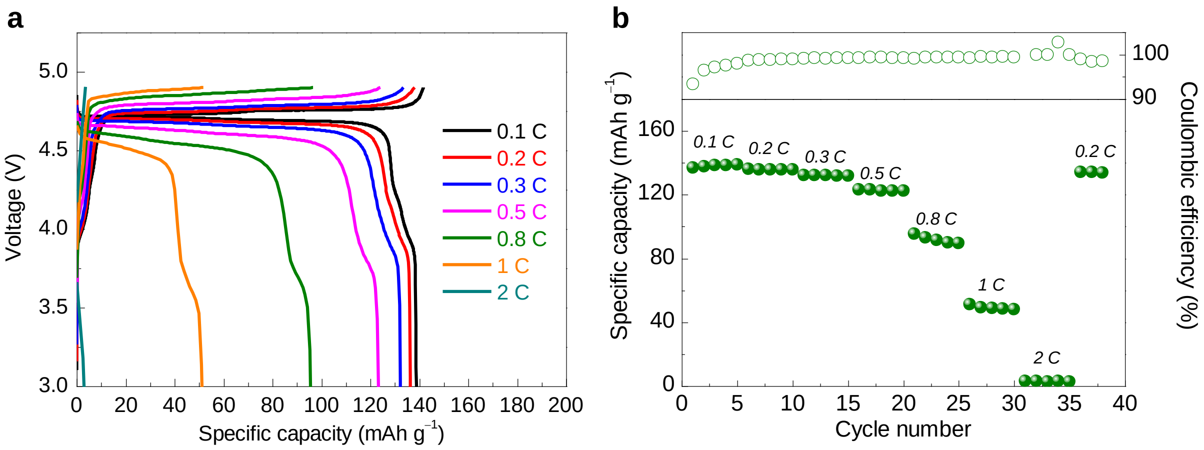

Supplementary Fig. 15 (a) Galvanostatic charge-discharge profiles at different C- rates and (b) rate performance analysis of the Li||$LiNi_{0.5}Mn_{1.5}O_4$ cell cycled with 1 M-LPF-DMDOHD electrolyte (1 C corresponds to 142 mA g^{-1}).

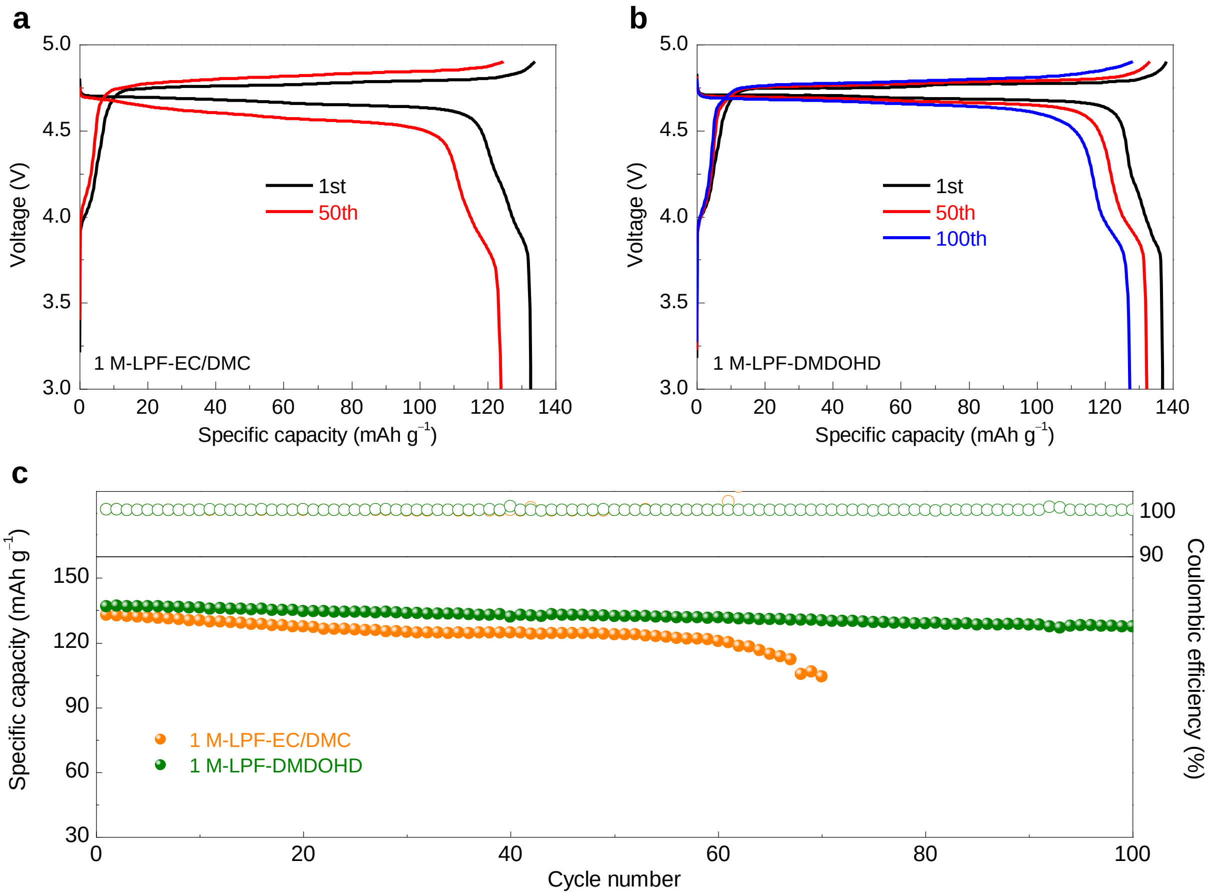

Supplementary Fig. 16 (a, b) Galvanostatic charge-discharge profiles at different cycle indices for Li||$LiNi_{0.5}Mn_{1.5}O_4$ cells cycled with (a) 1 M-LPF-EC/DMC and (b) 1 M-LPF-DMDOHD electrolytes at a rate of C/5. (c) Cycling stability of the Li||$LiNi_{0.5}Mn_{1.5}O_4$ cells in different electrolytes (C/5 cycling rate). The mass loading of the $LiNi_{0.5}Mn_{1.5}O_4$ positive electrode material is around 16 mg cm^{-2}, with an N/P ratio exceeding 50.

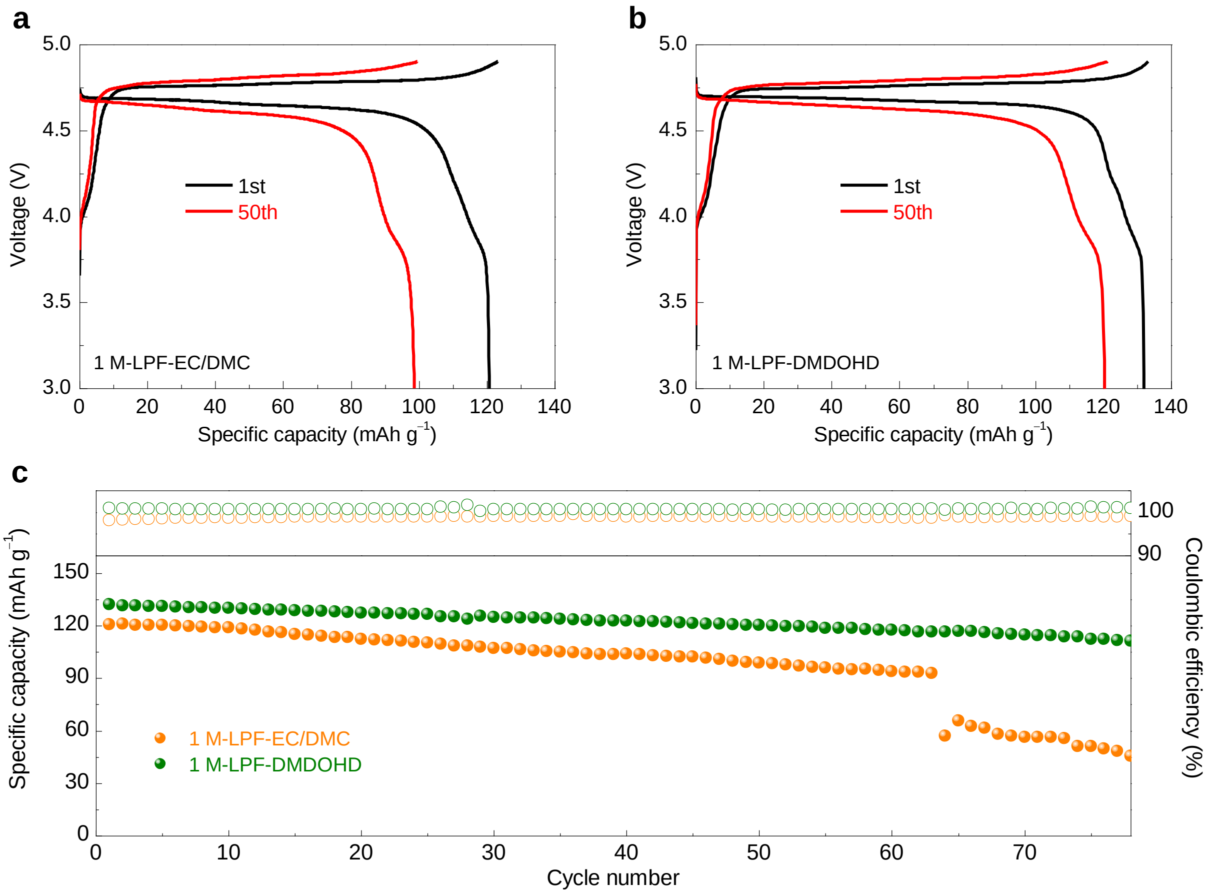

Supplementary Fig. 17 (a, b) Galvanostatic charge-discharge plots at different cycle indices for Li||$LiNi_{0.5}Mn_{1.5}O_4$ cells cycled with (a) 1 M-LPF-EC/DMC and (b) 1 M-LPF-DMDOHD electrolytes at a rate of C/5. (c) Cycling stability of the Li||$LiNi_{0.5}Mn_{1.5}O_4$ cells in different electrolytes (C/5 cycling rate). The mass loading of the $LiNi_{0.5}Mn_{1.5}O_4$ positive electrode material is around 16 mg cm^{-2}, with an N/P ratio of approximately 4.5.

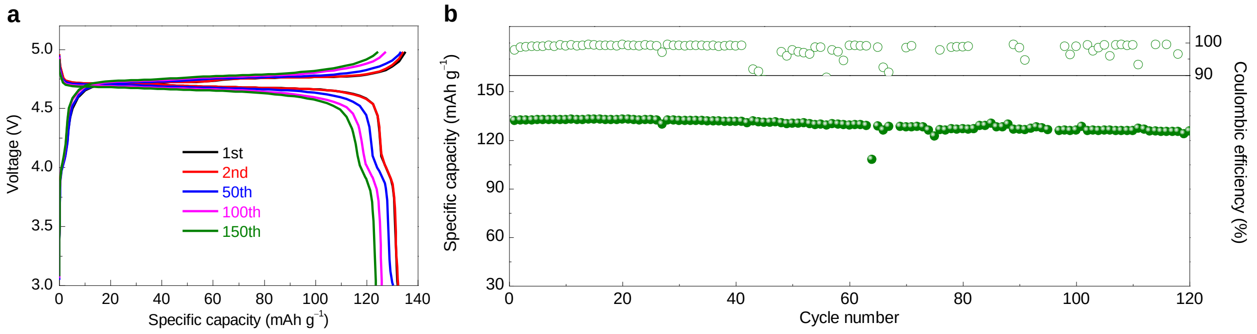

Supplementary Fig. 18 (a) Galvanostatic charge-discharge profiles and (b) cycling stability of the Li||$LiNi_{0.5}Mn_{1.5}O_4$ cell cycled in 1 M-LPF-DMDOHD electrolyte at a C-rate of C/3 with a working voltage window of 3−5 V (equivalent of a current density of 47.3 mA g^{-1}).

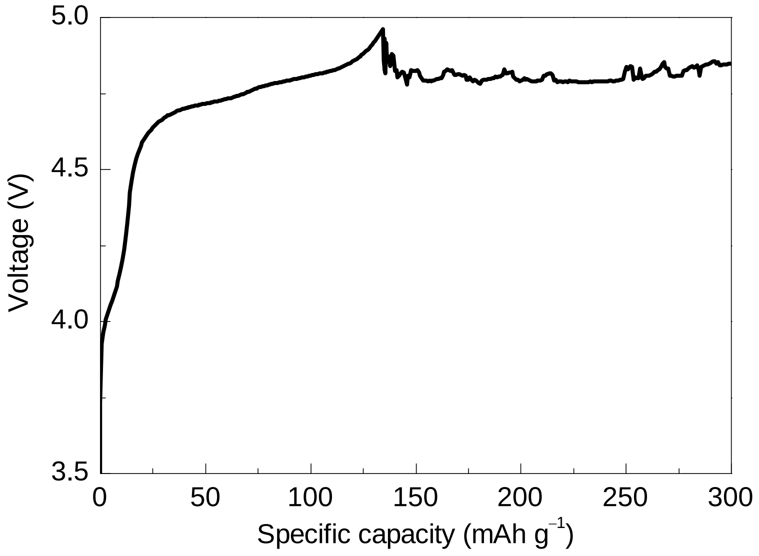

Supplementary Fig. 19 Galvanostatic charge(-discharge) plot of the Li||$LiNi_{0.5}Mn_{1.5}O_4$ cell cycled in 1 M-LPF-EC/DMC electrolytes at a C-rate of C/10 with a working voltage window of 3−5 V (equivalent of a current density of 47.3 mA g^{-1}).

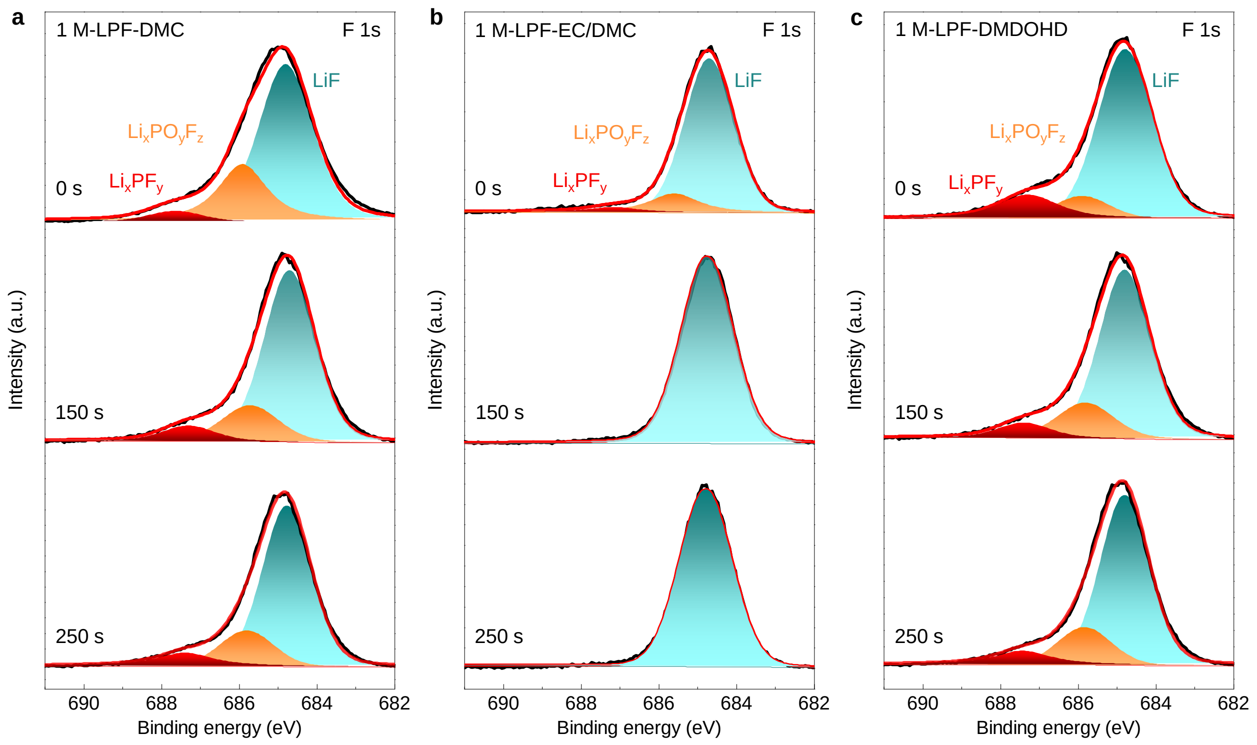

Supplementary Fig. 20 In depth profiling F 1s XPS spectra of Cu electrodes cycled in (a) 1 M-LPF-DMC, (b) 1 M-LPF-EC/DMC and (c) 1 M-LPF-DMDOHD electrolytes for various durations of Ar^+ sputtering (0, 150 and 250 s).

The samples were transferred using an air-tight transfer chamber with no exposure to ambient air. The Cu metal electrodes was taken from the Li||Cu cell after 10 cycles at 0.5 mA cm^{-2} for 1 h, ended with lithium plating in Cu surface.

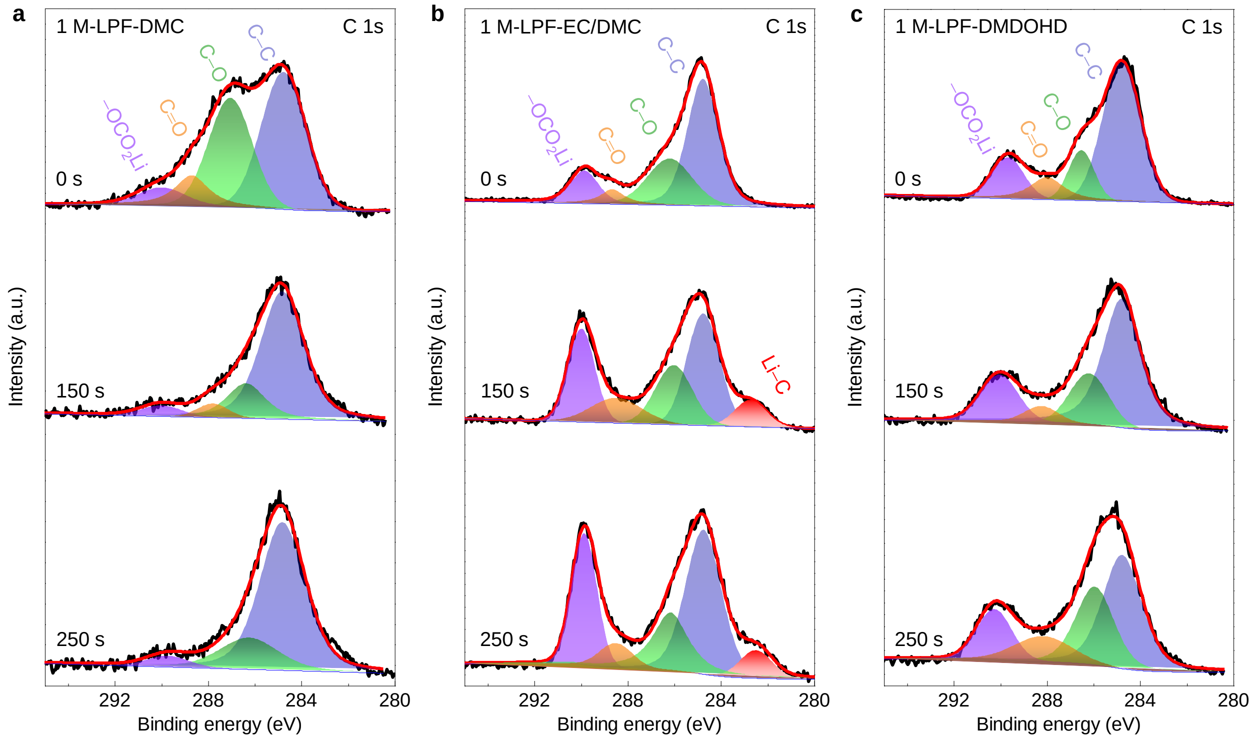

Supplementary Fig. 21 In depth profiling C 1s XPS spectra of Cu electrodes cycled in (a) 1 M-LPF-DMC, (b) 1 M-LPF-EC/DMC and (c) 1 M-LPF-DMDOHD electrolytes for various durations of Ar^+ sputtering (0, 150 and 250 s).

The samples were transferred using an air-tight transfer chamber with no exposure to ambient air. The Cu metal electrodes was taken from the Li||Cu cell after 10 cycles at 0.5 mA cm^{-2} for 1 h, ended with lithium plating in Cu surface.

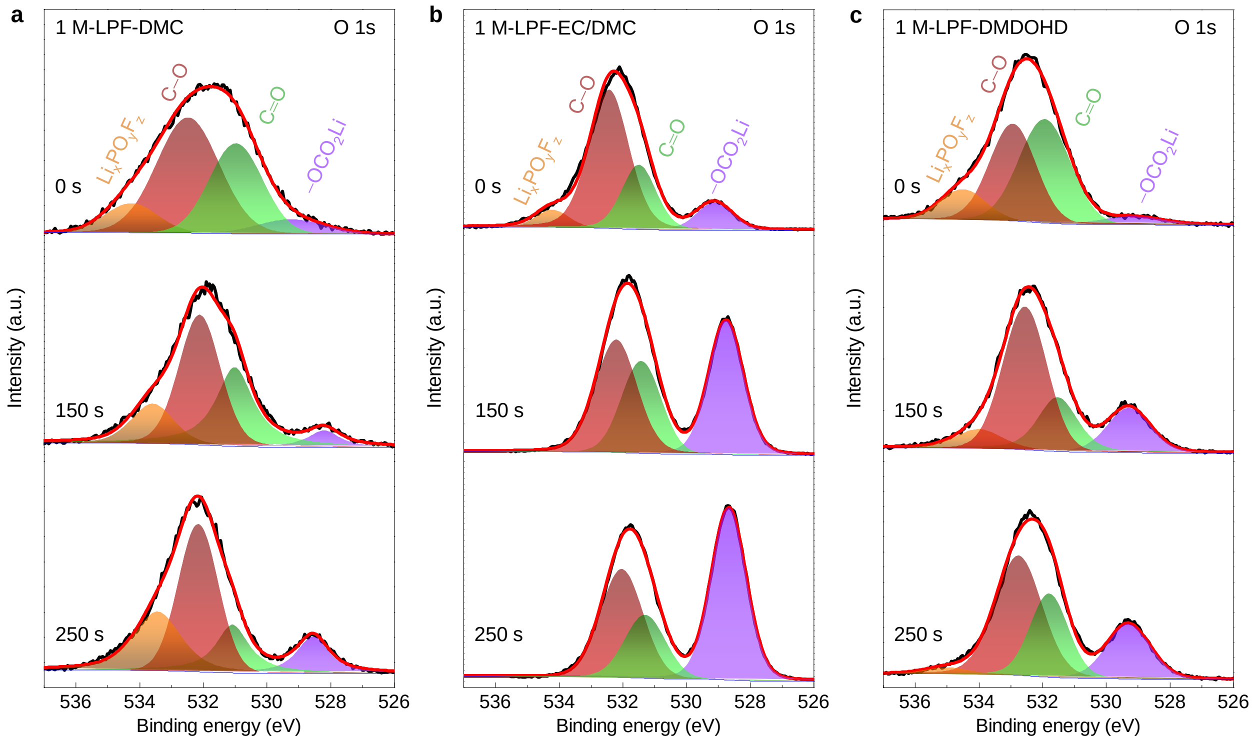

Supplementary Fig. 22 In depth profiling O 1s XPS spectra of Cu electrodes cycled in (a) 1 M-LPF-DMC, (b) 1 M-LPF-EC/DMC and (c) 1 M-LPF-DMDOHD electrolytes after various durations of Ar^+ sputtering. The samples were transferred using an air-tight transfer chamber with no exposure to ambient air. The Cu metal electrodes was taken from the Li||Cu cell after 10 cycles at 0.5 mA cm^{-2} for 1 h, ended with lithium plating in Cu surface.

Supplementary Table 12. SEI depth profile composition as determined from XPS analysis as function of electrolyte solvent.

Sample \ Component (%)	Etch time	C-C	C-O	C=O	$-OCO_2Li$	$Li_xPO_yF_z$	LiF	$Li_xP_yF_z$
DMC	0 s	5.53	12.09	7.24	2.31	19.92	47.03	5.88
	150 s	2.99	6.59	4.61	1.11	16.83	60.33	7.54
	250 s	3.48	6.12	2.53	2.10	17.07	61.29	7.41
EC/DMC	0 s	10.75	21.86	10.06	8.24	7.86	39.06	2.17
	150 s	3.47	21.38	16.76	27.04	-	31.35	-
	250 s	4.18	19.69	13.45	29.43	-	33.25	-
DMDOHD	0 s	9.93	12.03	10.80	3.43	10.82	46.36	6.63
	150 s	6.72	13.90	5.98	7.10	11.85	49.49	4.96
	250 s	4.78	18.35	13.11	10.54	8.19	40.94	4.09

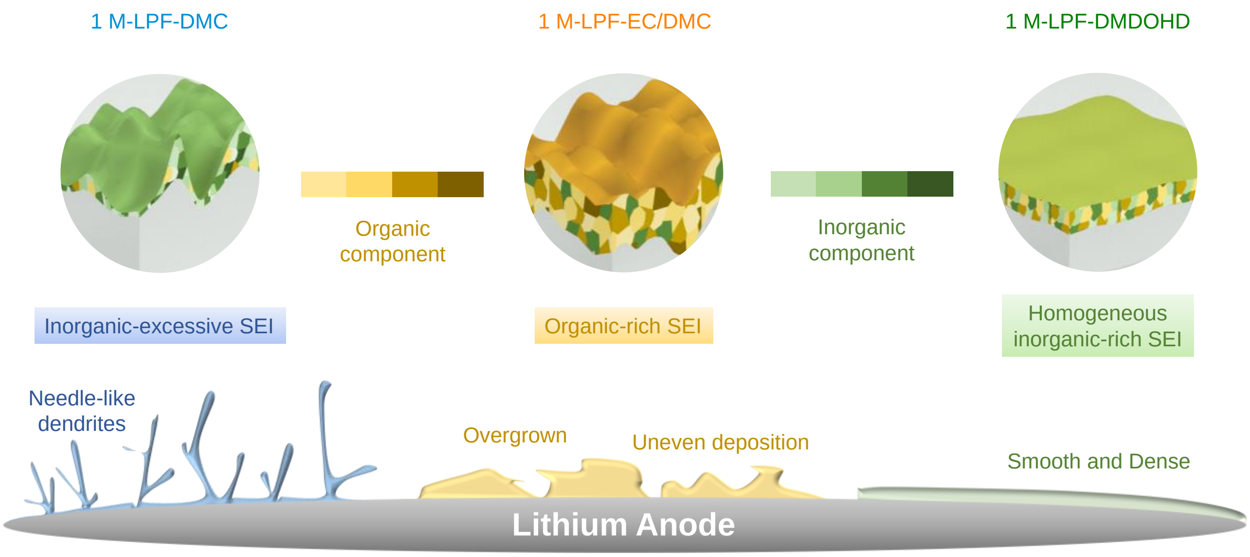

Supplementary Fig. 23 Schematics showing the chemical composition and morphology of the solid-electrolyte interphase (SEI) generated on the cycled lithium metal negative electrode surface in different electrolytes.

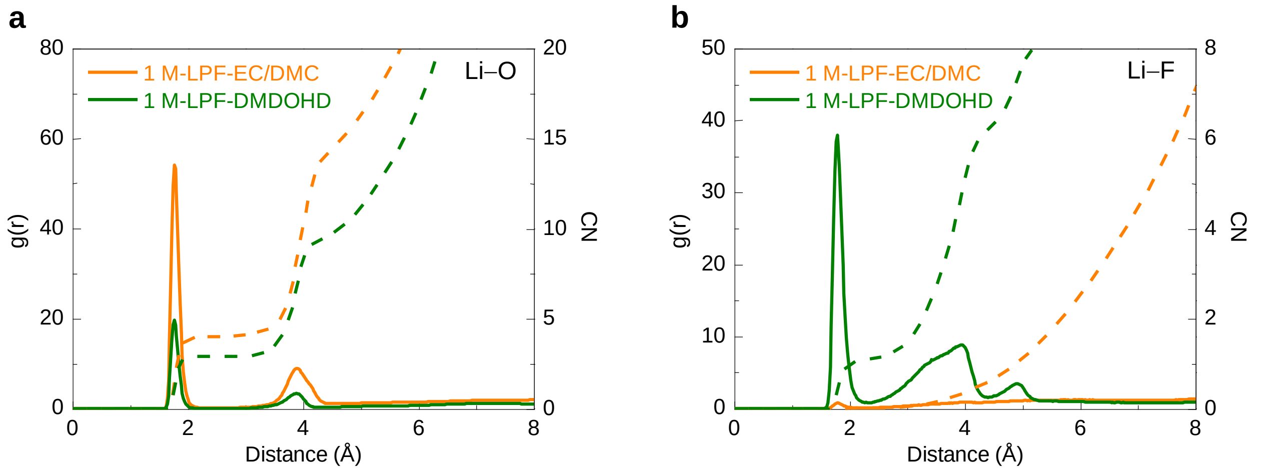

Supplementary Fig. 24 Radial distribution functions (g(r), solid lines) and coordination numbers (CN, dashed lines) of Li^+ with (a) solvent (Li–O) and (b) anion (Li–F) in 1 M-LPF-EC/DMC and 1 M-LPF-DMDOHD electrolyte formulations.

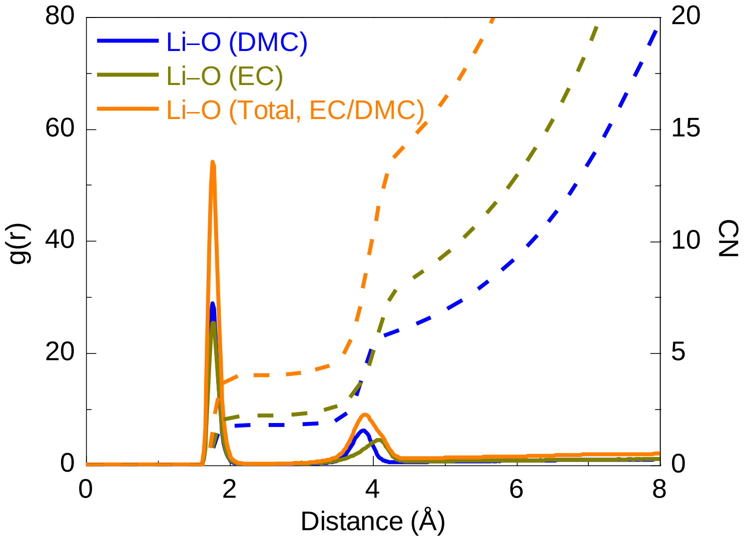

Supplementary Fig. 25 Comparison of radial distribution functions (g(r), solid lines) and coordination numbers (CN, dashed lines) of Li^+ with DMC and EC in 1 M-LPF-EC/DMC electrolyte formulation. In the 1 M-LPF-EC/DMC electrolyte system, the average CN values of Li^+ with EC and DMC are≈2.2 and ≈1.8, respectively.

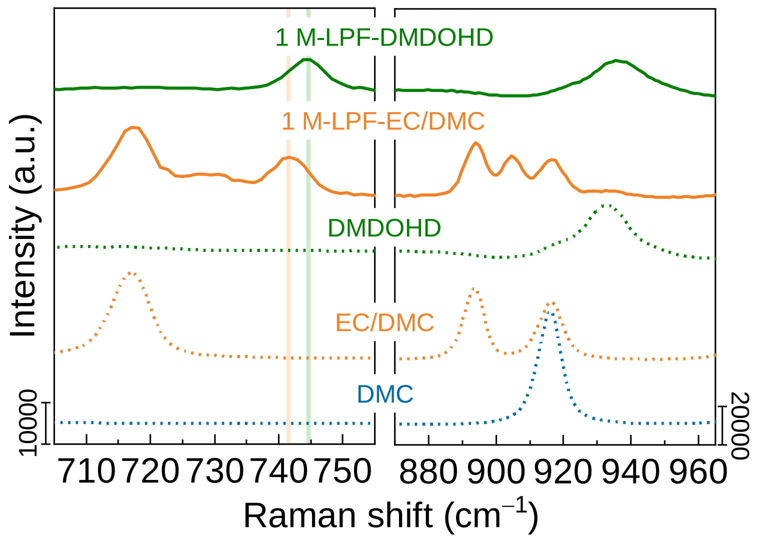

Supplementary Fig. 26 Raman spectra of 1 M-LPF-EC/DMC and 1 M-LPF-DMDOHD electrolytes.

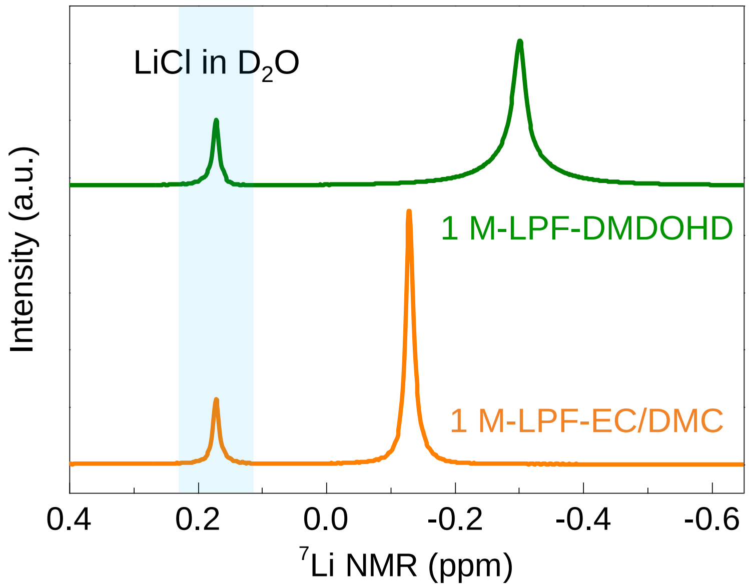

Supplementary Fig. 27 ^{7}Li NMR spectra of 1 M-LPF-EC/DMC and 1 M-LPF-DMDOHD electrolytes.

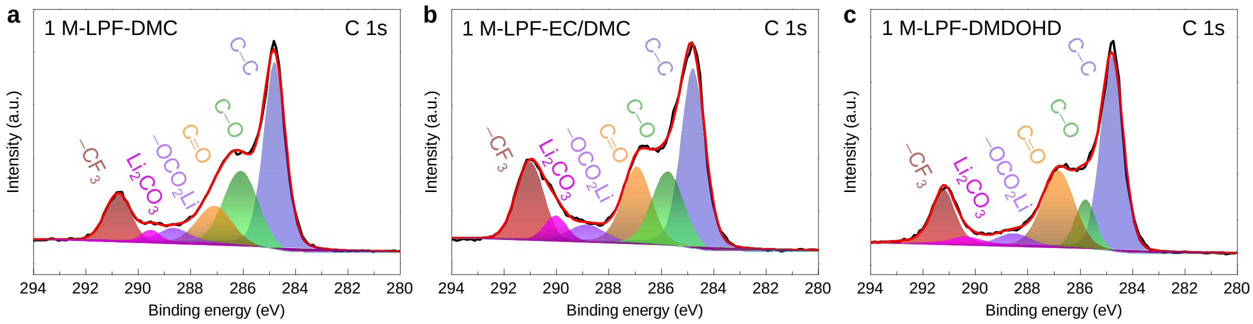

Supplementary Fig. 28 C 1s XPS spectra of the $LiNi_{0.5}Mn_{1.5}O_4$ electrode surface cycled in (a) 1 M-LPF-DMC, (b) 1 M-LPF-EC/DMC and (c) 1 M-LPF-DMDOHD electrolytes.

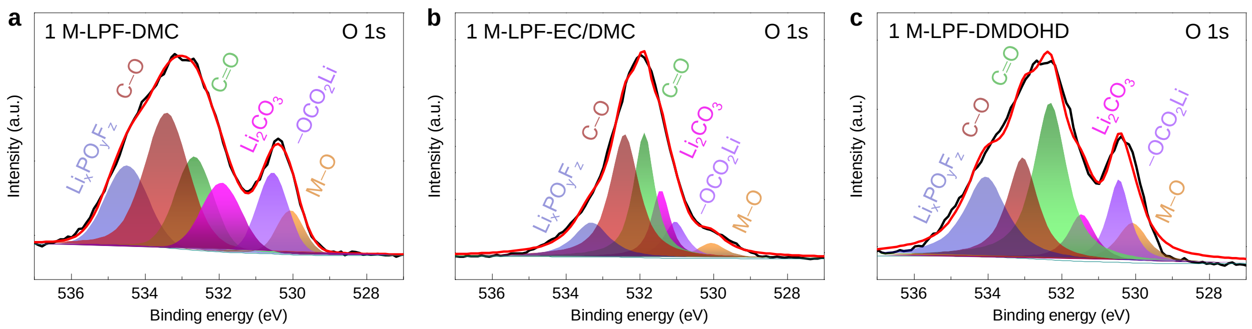

Supplementary Fig. 29 O 1s XPS spectra of the $LiNi_{0.5}Mn_{1.5}O_4$ electrode surface cycled in (a) 1 M-LPF-DMC, (b) 1 M-LPF-EC/DMC and (c) 1 M-LPF-DMDOHD electrolytes.

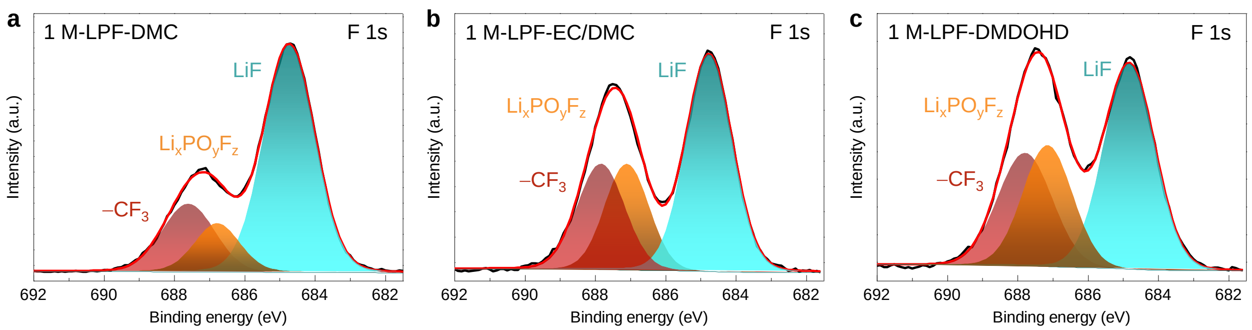

Supplementary Fig. 30 F 1s XPS spectra of the $LiNi_{0.5}Mn_{1.5}O_4$ electrode surface cycled in (a) 1 M-LPF-DMC, (b) 1 M-LPF-EC/DMC and (c) 1 M-LPF-DMDOHD electrolytes.

Supplementary Table 13. CEI composition at the surface of $LiNi_{0.5}Mn_{1.5}O_4$ electrodes cycled in electrolytes with different solvents, as determined from XPS data analysis.

Component (%) / Sample	$-CF_3$	M-O	Li_2CO_3	$-OCO_2Li$	C=O	C-O	C-C	$Li_xPO_yF_Z$	LiF
**DMC**	14.08	1.59	6.09	3.89	6.29	10.36	20.88	10.25	26.57
**EC/DMC**	15.88	1.13	7.91	5.63	14.39	13.54	16.87	9.72	14.93
**DMDOHD**	16.36	1.35	2.11	3.47	14.65	9.40	23.14	12.22	17.30

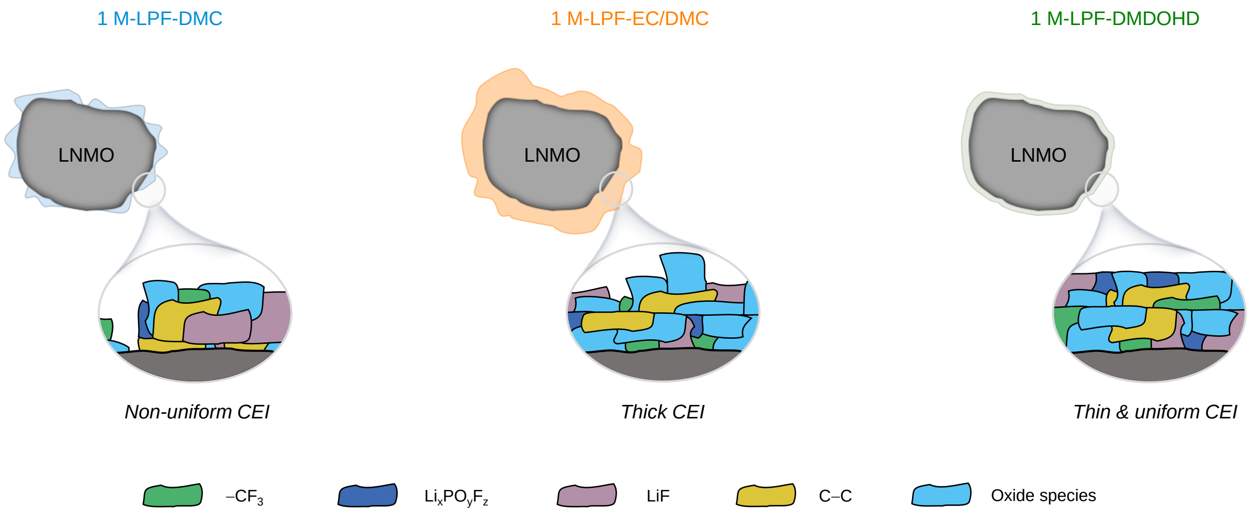

Supplementary Fig. 31 Schematics showing the chemical composition and morphological structure of the cathode-electrolyte interphase (CEI) generated on the cycled $LiNi_{0.5}Mn_{1.5}O_4$ positive electrode materials in different electrolytes.

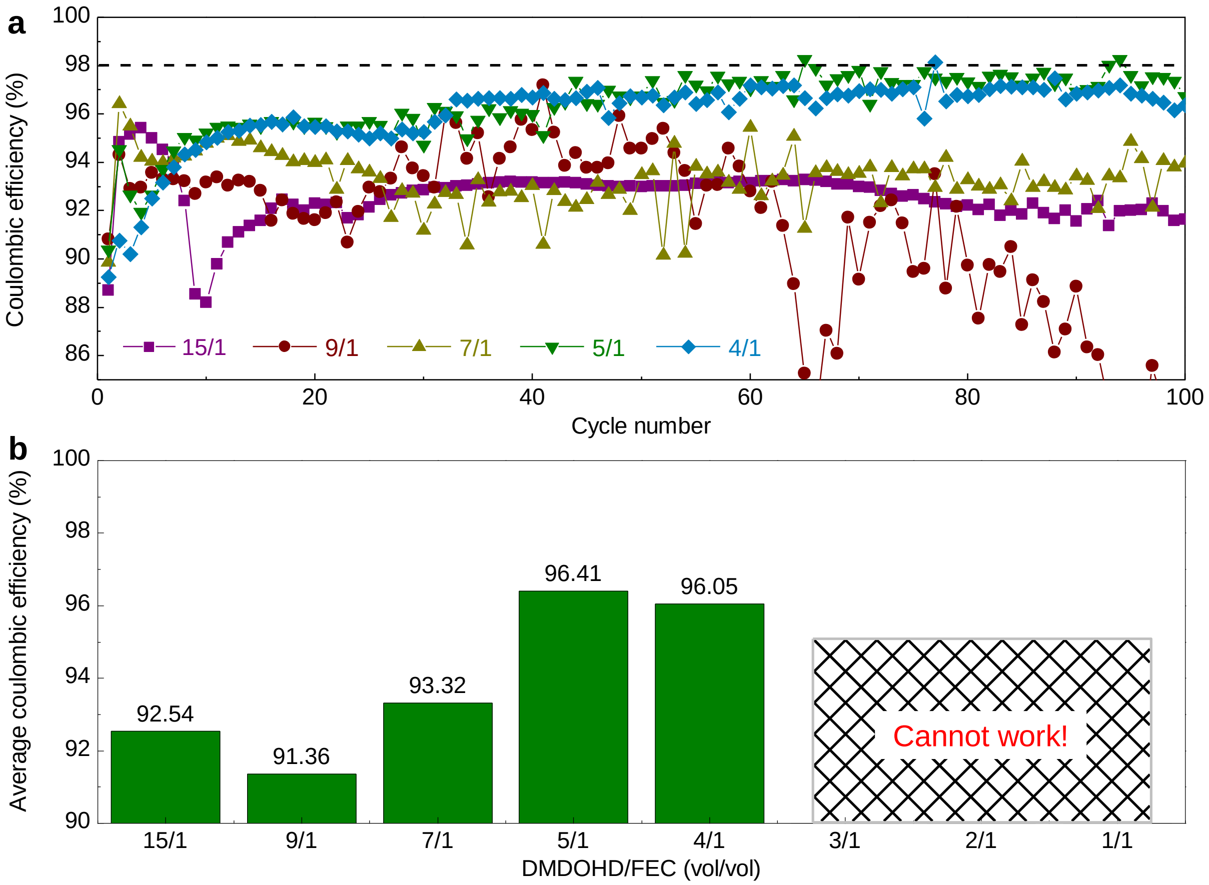

Supplementary Fig. 32 (a) Coulombic efficiency of the lithium plating/stripping process and (b) the corresponding average coulombic efficiency of the Li||Cu cell cycled in different DMDOHD/FEC-based electrolytes at a current density of 0.5 mA cm^{-2} with a cutoff capacity of 1 mAh cm^{-2}.

It is widely acknowledged that incorporating an appropriate quantity of FEC into the electrolyte facilitates the introduction of fluorine-containing components into the solid-electrolyte interface (SEI). The generation of fluorine-containing species contributes to the densification and robustness of the SEI, effectively mitigating the continuous side reactions between the electrolyte and lithium metal, as well as suppressing the growth of lithium dendrites. Consequently, this enhances the Coulombic efficiency of

lithium-metal batteries. However, Arumugam Manthiram *et al.* observed a decrease in the Coulombic efficiency of Li-metal batteries with excessively high FEC content.[56] This is primarily attributed to the high dielectric constant and polarity of FEC, similar to EC. FEC exhibits strong lithium-ion solvating ability, and the resulting solvation structure primarily exists as solvent-separated ion pairs (SSIPs). An increase in FEC content in the electrolyte leads to a reduction in the number of contact ion pairs (CIPs) and anion aggregates (AGGs) in the solvation structure. This ultimately results in an increase in organic species and a decrease in inorganic species in the SEI. However, as demonstrated in numerous studies,[19,57] the diffusion of Li ions through the SEI is hindered by the presence of organic species due to their higher bonding affinity, resulting in vertical lithium deposition and perforation the SEI. In addition, the organic SEI also possesses a porous structure that is not dense enough to completely block the diffusion of (fully, or partially solvated Li-ions) and thus further reaction between lithium metal and electrolyte solvent(s).[19] Consequently, the SEI properties formed in DMDOHD-based electrolytes with excessive FEC remain unsatisfactory in preventing lithium dendritic growth, which may account for the decreased lithium plating/stripping Coulombic efficiency.

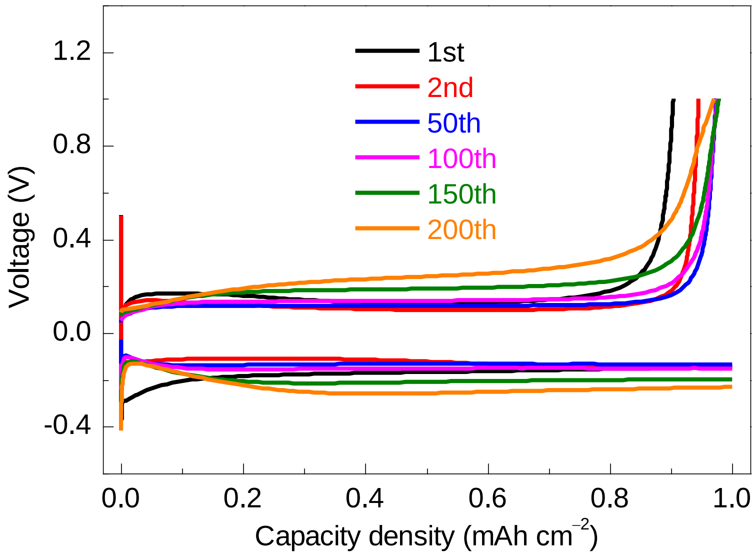

Supplementary Fig. 33 Lithium plating/stripping galvanostatic profiles measured in Li||Cu cells in 1 M-LPF-DMDOHD/FEC-5/1 (vol) electrolyte at a current density of 0.5 mA cm^{-2} with a cutoff capacity of 1 mAh cm^{-2}.

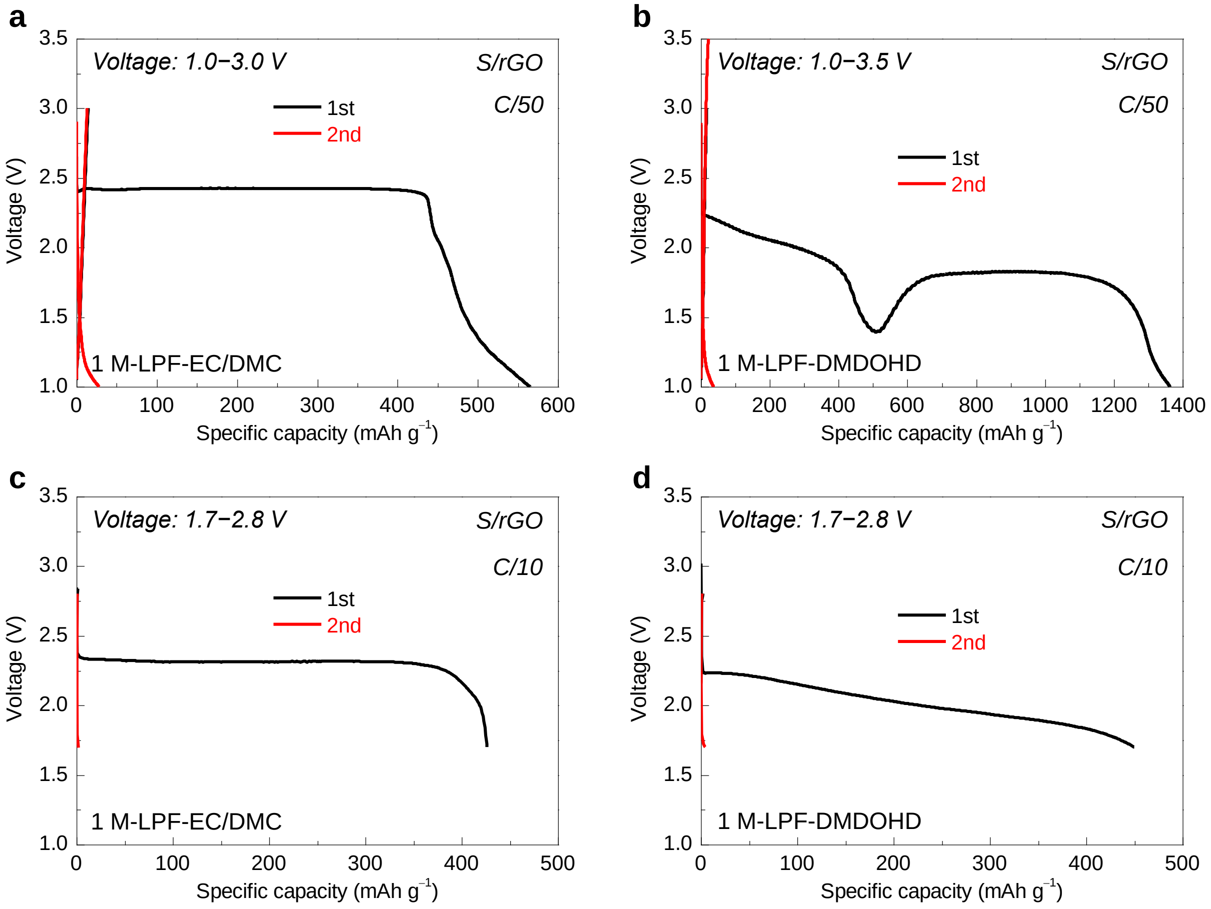

Supplementary Fig. 34 Galvanostatic charge-discharge plots at different cycle indices for the Li||S/rGO cells cycled with (a, c) 1 M-LPF-EC/DMC and (b, d) 1 M-LPF-DMDOHD electrolytes at a rate of (a, b) C/50 and (c, d) C/10 in different voltage windows (1 C corresponds to 1675 mA g^{-1}).

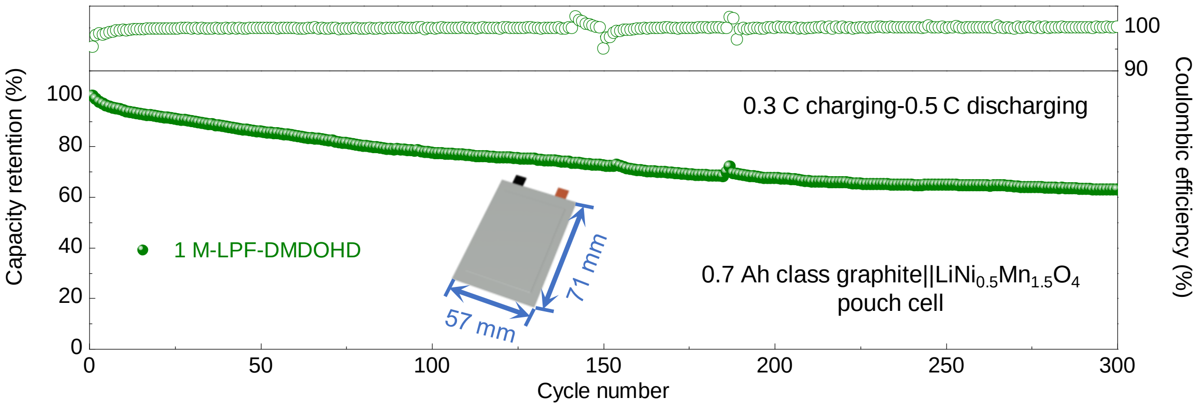

Supplementary Fig. 35 Cycling stability of the graphite||$LiNi_{0.5}Mn_{1.5}O_4$ pouch cell with 1 M-LPF-DMDOHD electrolyte (0.3 C charging-0.5 C discharging) at 25 °C. A 0.3 C constant current and 4.85 V constant voltage (CC-CV) mode was adopted for the charging sequence while a 0.5 C constant current (CC) mode was adopted for the discharging sequence.

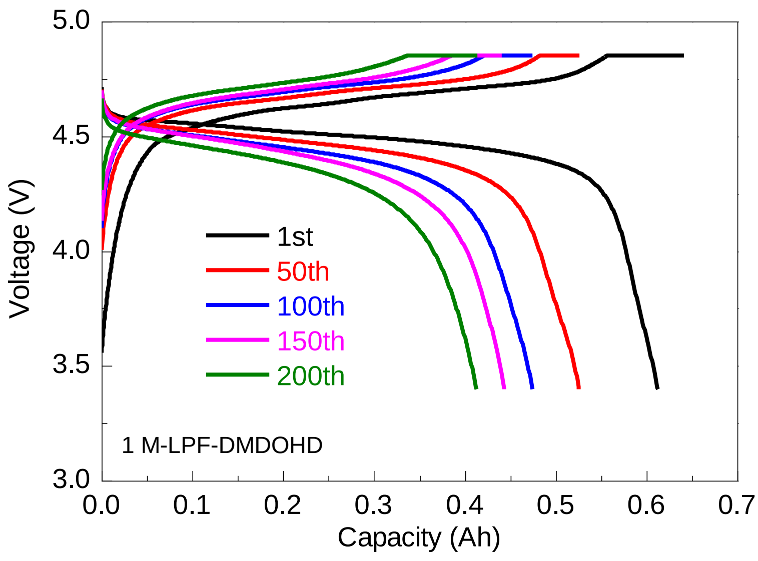

Supplementary Fig. 36 Selected voltage profiles of the graphite||$LiNi_{0.5}Mn_{1.5}O_4$ pouch cell with 1 M-LPF-DMDOHD electrolyte (0.3 C charging-0.5 C discharging) at 25 ºC. A 0.3 C constant current and 4.85 V constant voltage (CC-CV) mode was used for the charging sequence, while a 0.5 C constant current (CC) mode was adopted for the discharging sequence.

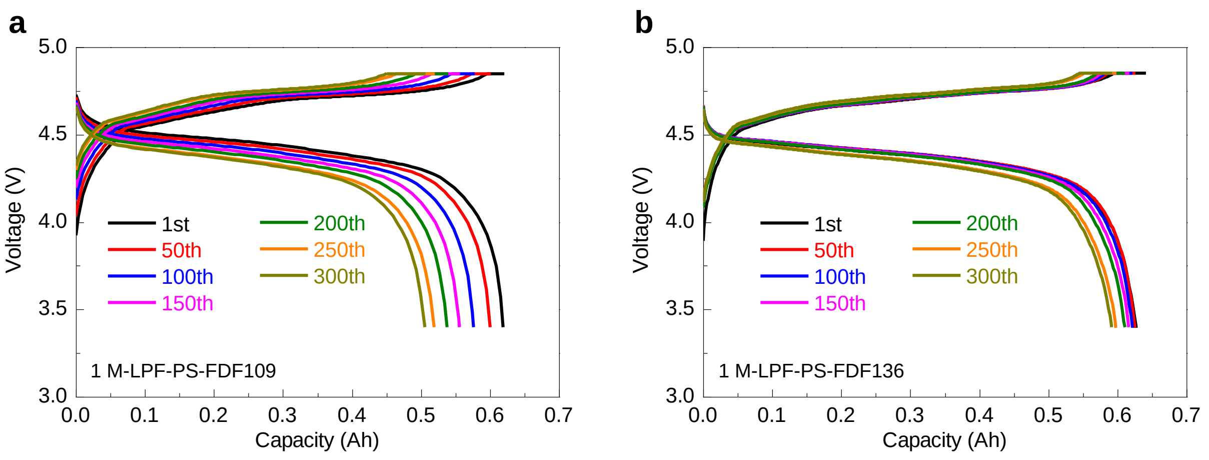

Supplementary Fig. 37 Selected voltage profiles of the graphite||$LiNi_{0.5}Mn_{1.5}O_4$ pouch cells (a) without and (b) with 30% volume ratio of DMDOHD in the FEC/FEMC baseline electrolyte (0.5 C charging-1 C discharging) at 25 °C. A 0.5 C constant current and 4.85 V constant voltage (CC-CV) mode was used for all the charging sequences while a 1 C constant current (CC) mode was applied for the discharging sequences. FEMC and PS represent 2,2,2-trifluoroethyl methyl carbonate and 1,3-propanesultone, respectively.

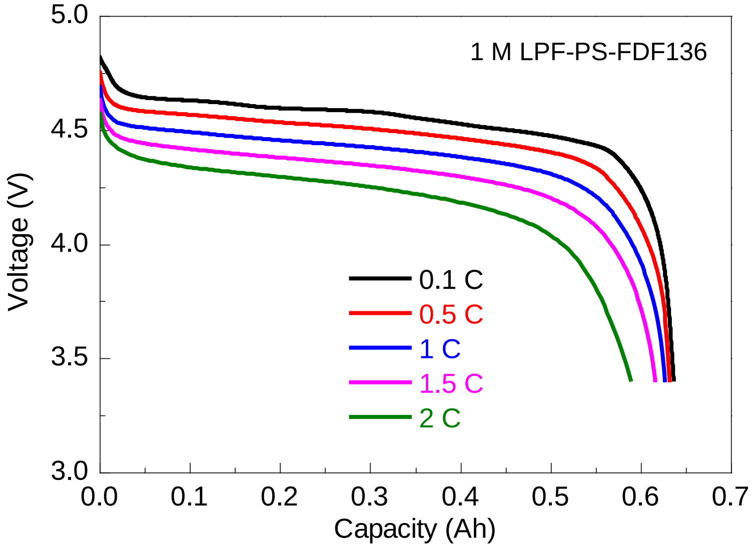

Supplementary Fig. 38 Rate capability galvanostatic charge-discharge profiles of the graphite||$LiNi_{0.5}Mn_{1.5}O_4$ pouch cell with a 30% volume ratio of DMDOHD in the FEC/FEMC-based electrolyte system at 25 ºC. A 0.3 C constant current and 4.85 V constant voltage (CC-CV) mode was adopted for all the charging sequences. Voltage profiles are plotted with respect to the cell capacity. FEMC and PS represent 2,2,2-trifluoroethyl methyl carbonate and 1,3-propanesultone, respectively.

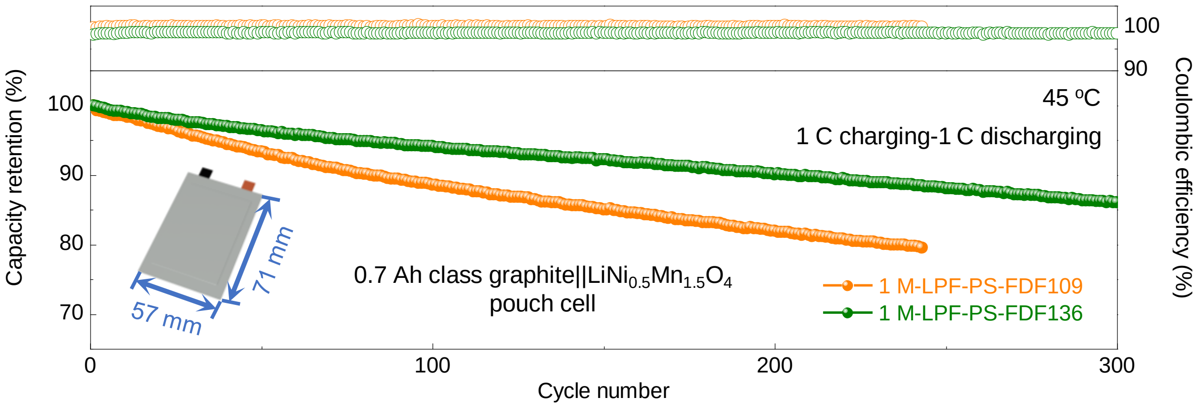

Supplementary Fig. 39 Cycling stability of the graphite||$LiNi_{0.5}Mn_{1.5}O_4$ pouch cells with different electrolytes (1 C charging-1 C discharging) at 45 °C. A 1 C constant current and 4.85 V constant voltage (CC-CV) mode was adopted for all the charging sequences while a 1 C constant current (CC) mode was adopted for all the discharging sequences. FEMC and PS represent 2,2,2-trifluoroethyl methyl carbonate and 1,3-propanesultone, respectively.

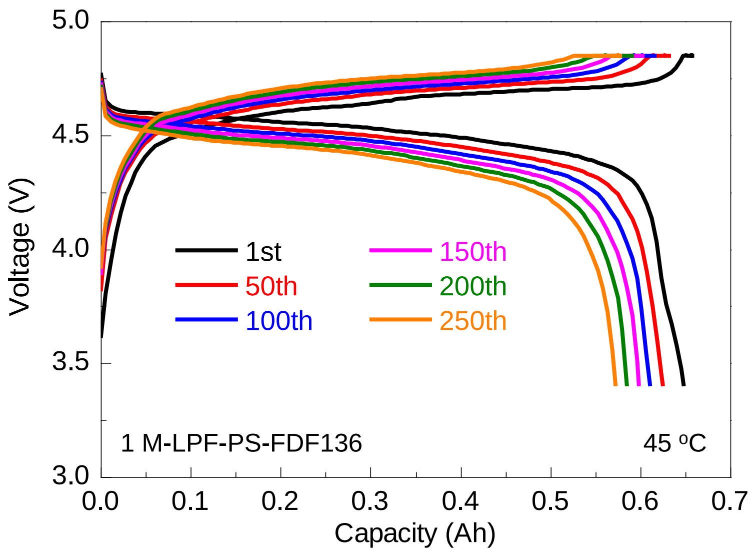

Supplementary Fig. 40 Selected voltage profiles of the graphite||$LiNi_{0.5}Mn_{1.5}O_4$ pouch cell with a 30% volume ratio of DMDOHD in the FEC/FEMC-based electrolyte system (1 C charging-1 C discharging) at 45 ºC. A 1 C constant current and 4.85 V constant voltage (CC-CV) mode was adopted for all the charging sequences while a 1 C constant current (CC) mode was adopted for all the discharging sequences. FEMC and PS represent 2,2,2-trifluoroethyl methyl carbonate and 1,3-propanesultone, respectively.

Reference

1 Li, Q. *et al.* Unraveling the New Role of an Ethylene Carbonate Solvation Shell in Rechargeable Metal Ion Batteries. *ACS Energy Lett.* **6**, 69–78 (2021).

2 Ren, X. *et al.* Guided Lithium Metal Deposition and Improved Lithium Coulombic Efficiency through Synergistic Effects of $LiAsF_6$ and Cyclic Carbonate Additives. *ACS Energy Lett.* **3**, 14–19 (2018).

3 Lee, T. J., Soon, J., Chae, S., Ryu, J. H. & Oh, S. M. A Bifunctional Electrolyte Additive for High-Voltage $LiNi_{0.5}Mn_{1.5}O_4$ Positive Electrodes. *ACS Appl. Mater. Interfaces* **11**, 11306–11316 (2019).

4 Luo, Y. *et al.* Enhanced electrochemical performance of $LiNi_{0.5}Mn_{1.5}O_4$ cathode using an electrolyte with 3-(1,1,2,2-tetrafluoroethoxy)-1,1,2,2-tetrafluoropropane. *J. Power Sources* **323**, 134–141 (2016).

5 Chen, Y.-Q. *et al.* An electrolyte additive with boron-nitrogen-oxygen alkyl group enabled stable cycling for high voltage $LiNi_{0.5}Mn_{1.5}O_4$ cathode in lithium-ion battery. *J. Power Sources* **477**, 228473 (2020).

6 Liu, B., Zhou, H., Yin, C., Guan, H. & Li, J. Enhanced electrochemical performance of $LiNi_{0.5}Mn_{1.5}O_4$ cathode by application of $LiPF_2O_2$ for lithium difluoro(oxalate)borate electrolyte. *Electrochim. Acta* **321**, 134690 (2019).

7 Xu, Y., Liu, J., Zhou, L., Zeng, L. & Yang, Z. FEC as the additive of 5V electrolyte and its electrochemical performance for $LiNi_{0.5}Mn_{1.5}O_4$. *J. Electroanal. Chem.* **791**, 109–116 (2017).

8 Zheng, X. *et al.* Exploring high-voltage fluorinated carbonate electrolytes for $LiNi_{0.5}Mn_{1.5}O_4$ cathode in Li-ion batteries. *J. Energy Chem.* **42**, 62–70 (2020).

9 Bolloju, S., Chiou, C.-Y., Vikramaditya, T. & Lee, J.-T. (Pentafluorophenyl)diphenylphosphine as a dual-functional electrolyte additive for $LiNi_{0.5}Mn_{1.5}O_4$ cathodes in high-voltage lithium-ion batteries. *Electrochim. Acta* **299**, 663–671 (2019).

10 Tan, C. *et al.* Optimizing interphase structure to enhance electrochemical performance of high voltage $LiNi_{0.5}Mn_{1.5}O_4$ cathode via anhydride additives. *Chem. Eng. J.* **410**, 128422 (2021).

11 Mai, S., Xu, M., Liao, X., Xing, L. & Li, W. Improving cyclic stability of lithium nickel manganese oxide cathode at elevated temperature by using dimethyl phenylphosphonite as electrolyte additive. *J. Power Sources* **273**, 816–822 (2015).

12 Doi, T., Masuhara, R., Hashinokuchi, M., Shimizu, Y. & Inaba, M. Concentrated $LiPF_6$/PC electrolyte solutions for 5-V $LiNi_{0.5}Mn_{1.5}O_4$ positive electrode in lithium-ion batteries. *Electrochim. Acta* **209**, 219–224 (2016).

13 Shang, H. *et al.* Improving the Cyclic Stability of $LiNi_{0.5}Mn_{1.5}O_4$ at High Cutoff Voltage by Using Pyrene as a Novel Additive. *Energy Technol.* **8**, 2000671 (2020).

14 Huang, Y. *et al.* Modifying the Cathode–Electrolyte Interphase by Sulfone-Based Additive to Enhance the Electrochemical Performance of $LiNi_{0.5}Mn_{1.5}O_4$. *ACS Appl. Energy Mater.* **5**, 639–647 (2022).

15 Sun, D. *et al.* Forming a Stable CEI Layer on $LiNi_{0.5}Mn_{1.5}O_4$ Cathode by the Synergy Effect of FEC and HDI. *J. Electrochem. Soc.* **165**, A2032 (2018).

16 Piao, N. *et al.* Countersolvent Electrolytes for Lithium-Metal Batteries. *Adv. Energy Mater.* **10**, 1903568 (2020).

17 Li, S. *et al.* Synergistic Dual-Additive Electrolyte Enables Practical Lithium-Metal Batteries. *Angew. Chem. Int. Ed.* **59**, 14935–14941 (2020).

18 Zhang, Q. *et al.* Highly safe and cyclable Li-metal batteries with vinylethylene carbonate electrolyte. *Nano Energy* **74**, 104860 (2020).

19 Liu, S. *et al.* An Inorganic-Rich Solid Electrolyte Interphase for Advanced Lithium-Metal Batteries in Carbonate Electrolytes. *Angew. Chem. Int. Ed.* **60**, 3661–3671 (2021).

20 Yang, Y. *et al.* Vinyl Ethylene Carbonate as an Effective SEI-Forming Additive in Carbonate-Based Electrolyte for Lithium-Metal Anodes. *ACS Appl. Mater. Interfaces* **11**, 6118–6125 (2019).

21 Zhang, Y., Zhong, Y., Shi, Q., Liang, S. & Wang, H. Cycling and Failing of Lithium Metal Anodes in Carbonate Electrolyte. *J. Phys. Chem. C* **122**, 21462–21467 (2018).

22 Zhang, Y. *et al.* Formation and Evolution of Lithium Metal Anode–Carbonate Electrolyte Interphases. *ACS Mater. Lett.* **1**, 254–259 (2019).

23 Jiang, H. *et al.* Lithium dendrite suppression by facile interfacial barium engineering for stable 5 V-class lithium metal batteries with carbonate-based electrolyte. *Chem. Eng. J.* **414**, 128928 (2021).

24 Xiao, P. *et al.* High-Performance Lithium Metal Batteries with a Wide Operating Temperature Range in Carbonate Electrolyte by Manipulating Interfacial Chemistry. *ACS Energy Lett.* **6**, 3170–3179 (2021).

25 Ren, X. *et al.* Enabling High-Voltage Lithium-Metal Batteries under Practical Conditions. *Joule* **3**, 1662–1676 (2019).

26 Su, C.-C. *et al.* Cyclic carbonate for highly stable cycling of high voltage lithium metal batteries. *Energy Storage Mater.* **17**, 284–292 (2019).

27 Piao, N. *et al.* Lithium Metal Batteries Enabled by Synergetic Additives in Commercial Carbonate Electrolytes. *ACS Energy Lett.* **6**, 1839–1848 (2021).

28 Guo, Z. *et al.* Cationic Size Effect Promoting Dissolution of Nitrate Anion in Ester Electrolyte for Lithium–Metal Batteries. *ACS Energy Lett.* **7**, 569–576 (2022).

29 Yoshitake, H. in *Lithium-Ion Batteries: Science and Technologies* (eds Masaki Yoshio, Ralph J. Brodd, & Akiya Kozawa) 343–366 (Springer New York, 2009).

30 Liu, J. *et al.* Tuning Interphase Chemistry to Stabilize High-Voltage $LiCoO_2$ Cathode Material via Spinel Coating. *Angew. Chem. Int. Ed.* **61**, e202207000 (2022).

31 Yu, L. *et al.* Preferential Adsorption of Solvents on the Cathode Surface of Lithium Ion Batteries. *Angew. Chem. Int. Ed.* **52**, 5753–5756 (2013).

32 Borodin, O. *et al.* Modeling Insight into Battery Electrolyte Electrochemical Stability and Interfacial Structure. *Acc. Chem. Res.* **50**, 2886–2894 (2017).

33 Vatamanu, J., Borodin, O. & Smith, G. D. Molecular Dynamics Simulation Studies of the Structure of a Mixed Carbonate/$LiPF_6$ Electrolyte near Graphite Surface as a Function of Electrode Potential. *J. Phys. Chem. C* **116**, 1114–1121 (2012).

34 Jow, T. R., Xu, K., Borodin, O. & Ue, M. *Electrolytes for lithium and lithium-ion batteries*. Vol. 58 (Springer, 2014).

35 Borodin, O., Wu, F. & Yushin, G. In situ surface protection for enhancing stability and performance of conversion-type cathodes. *MRS Energy Sustain.* **4**, E9 (2017).

36 Borodin, O., Behl, W. & Jow, T. R. Oxidative Stability and Initial Decomposition Reactions of Carbonate, Sulfone, and Alkyl Phosphate-Based Electrolytes. *J. Phys. Chem. C* **117**, 8661–8682 (2013).

37 Fan, X. & Wang, C. High-voltage liquid electrolytes for Li batteries: progress and perspectives. *Chem. Soc. Rev.* **50**, 10486–10566 (2021).

38 Fan, X. *et al.* Non-flammable electrolyte enables Li-metal batteries with aggressive cathode chemistries. *Nat. Nanotechnol.* **13**, 715–722 (2018).

39 Xu, K. Electrolytes and Interphases in Li-Ion Batteries and Beyond. *Chem. Rev.* **114**, 11503–11618 (2014).

40 Li, R. *et al.* Unitized Configuration Design of Thermally Stable Composite Polymer Electrolyte for Lithium Batteries Capable of Working Over a Wide Range of Temperatures. *Adv. Eng. Mater.* **21**, 1900055 (2019).

41 Ma, F. *et al.* Solid Polymer Electrolyte Based on Polymerized Ionic Liquid for High Performance All-Solid-State Lithium-Ion Batteries. *ACS Sustain. Chem. Eng.* **7**, 4675–4683 (2019).

42 Zhai, H. *et al.* A Flexible Solid Composite Electrolyte with Vertically Aligned and Connected Ion-Conducting Nanoparticles for Lithium Batteries. *Nano Lett.* **17**, 3182–3187 (2017).

43 Thangadurai, V. & Weppner, W. $Li_6ALa_2Ta_2O_{12}$ (A = Sr, Ba): Novel Garnet-Like Oxides for Fast Lithium Ion Conduction. *Adv. Funct. Mater.* **15**, 107–112 (2005).

44 Murugan, R., Thangadurai, V. & Weppner, W. Fast Lithium Ion Conduction in Garnet-Type $Li_7La_3Zr_2O_{12}$. *Angew. Chem. Int. Ed.* **46**, 7778–7781 (2007).

45 Gong, Y. *et al.* Lithium-ion conductive ceramic textile: A new architecture for flexible solid-state lithium metal batteries. *Mater. Today* **21**, 594–601 (2018).

46 Li, Z., Sha, W.-X. & Guo, X. Three-Dimensional Garnet Framework-Reinforced Solid Composite Electrolytes with High Lithium-Ion Conductivity and Excellent Stability. *ACS Appl. Mater. Interfaces* **11**, 26920–26927 (2019).

47 Inaguma, Y. *et al.* High ionic conductivity in lithium lanthanum titanate. *Solid State Commun.* **86**, 689–693 (1993).

48 Le Van-Jodin, L., Ducroquet, F., Sabary, F. & Chevalier, I. Dielectric properties, conductivity and Li+ ion motion in LiPON thin films. *Solid State Ion.* **253**, 151–156 (2013).

49 Sun, J., Forsyth, M. & MacFarlane, D. R. Room-Temperature Molten Salts Based on the Quaternary Ammonium Ion. *J. Phys. Chem. B* **102**, 8858–8864 (1998).

50 Guzmán-González, G. *et al.* Lithium Borate Ionic Liquids as Single-Component Electrolytes for Batteries. *Adv. Energy Mater.* **13**, 2202974 (2023).

51 Bouchet, R. *et al.* Single-ion BAB triblock copolymers as highly efficient electrolytes for lithium-metal batteries. *Nat. Mater.* **12**, 452–457 (2013).

52 Zheng, J. *et al.* In-situ polymerization with dual-function electrolyte additive toward future lithium metal batteries. *Mater. Today Energy* **26**, 100984 (2022).

53 Li, Q. *et al.* Engineering a High-Voltage Durable Cathode/Electrolyte Interface for All-Solid-State Lithium Metal Batteries via In Situ Electropolymerization. *ACS Appl. Mater. Interfaces* **14**, 21018–21027 (2022).

54 Chen, X. *et al.* An Ultra-Thin Crosslinked Carbonate Ester Electrolyte for 24 V Bipolar Lithium-Metal Batteries. *J. Electrochem. Soc.* **169**, 090509 (2022).

55 Duan, H. *et al.* Dendrite-Free Li-Metal Battery Enabled by a Thin Asymmetric Solid Electrolyte with Engineered Layers. *J. Am. Chem. Soc.* **140**, 82–85 (2018).

56 Yi, M., Su, L. & Manthiram, A. Tuning and understanding the solvent ratios of localized saturated electrolytes for lithium-metal batteries. *J. Mater. Chem. A* **11**, 11889–11902 (2023).

57 Fang, C. *et al.* Quantifying inactive lithium in lithium metal batteries. *Nature* **572**, 511–515 (2019).
