# Supplementary material for: A dicarbonate solvent electrolyte for high performance 5 V-Class Lithium-based batteries
Source: Nat Commun. 2024 Jan 15;15:536. doi: 10.1038/s41467-024-44858-3 (PMC10789778; doi:10.1038/s41467-024-44858-3)
Supplement: Supplementary file 3 — Description of Additional Supplementary Files [file 41467_2024_44858_MOESM3_ESM.pdf]

Description of Additional Supplementary Files

File Name: **Supplementary Movie 1**
Description**:** Flammability tests for the conventional 1 M-LPF-EC/DMC electrolyte in the conditions of ignition of a suspended separator impregnated with electrolyte using a gentle flame.

File Name: **Supplementary Movie 2**
Description: Flammability tests for the 1 M-LPF-DMDOHD electrolyte in the conditions of ignition of a suspended separator impregnated with electrolyte using a gentle flame.

File Name: **Supplementary Movie 3**
Description: Flammability tests for the 1 M-LPF-DMDOHD electrolyte in the conditions of ignition of a suspended separator impregnated with electrolyte using a medium strength flame.

File Name: **Supplementary Movie 4**
Description: Flammability tests for the 1 M-LPF-DMDOHD electrolyte in the conditions of ignition of a suspended separator impregnated with electrolyte using a violent flame.

File Name: **Supplementary Movie 5**
Description: Flammability tests for the conventional 1 M-LPF-EC/DMC electrolyte (0.119 g) in the conditions of ignition of a suspended separator impregnated with electrolyte using a medium strength flame.

File Name: **Supplementary Movie 6**
Description: Flammability tests for the 1 M-LPF-DMDOHD electrolyte (0.130 g) in the conditions of ignition of a suspended separator impregnated with electrolyte using a medium strength flame.

File Name: **Supplementary Movie 7**
Description: Flammability tests for the conventional 1 M-LPF-EC/DMC electrolyte (0.726 g) in the condition of ignition of an electrolyte in a stainless-steel coin cell case.

File Name: **Supplementary Movie 8**
Description: Flammability tests for the 1 M-LPF-DMDOHD electrolyte (0.754 g) in the condition of ignition of an electrolyte in a stainless-steel coin cell case.

File Name: **Supplementary Movie 9**
Description: Flammability tests for the conventional 1 M-LPF-EC/DMC electrolyte (0.704 g) in the condition of ignition of a separator immersed in electrolyte in a stainless-steel coin cell case.

File Name: **Supplementary Movie 10**
Description: Flammability tests for the 1 M-LPF-DMDOHD electrolyte (0.754 g) in the condition of ignition of a separator immersed in electrolyte in a stainless-steel coin cell case.

File Name: **Supplementary Movie 11**

Description: In-situ optical microscopy observation of dynamic lithium deposition at a current density of 1 mA cm−2 in the 1 M-LPF-DMC electrolyte.

File Name: **Supplementary Movie 12**
Description: In-situ optical microscopy observation of dynamic lithium deposition at a current density of 1 mA cm−2 in the 1 M-LPF-EC/DMC electrolyte.

File Name: **Supplementary Movie 13**
Description: In-situ optical microscopy observation of dynamic lithium deposition at a current density of 1 mA cm−2 in the 1 M-LPF-DMDOHD electrolyte.
